# Na_V_1.5 knockout in iPSCs: a novel approach to study Na_V_1.5 variants in a human cardiomyocyte environment

**DOI:** 10.1038/s41598-021-96474-6

**Published:** 2021-08-25

**Authors:** Marion Pierre, Mohammed Djemai, Hugo Poulin, Mohamed Chahine

**Affiliations:** 1 CERVO Brain Research Center, 2601, de La Canardière, Quebec City, QC G1J 2G3 Canada; 2 Department of Medicine, Faculty of Medicine, Université Laval, Quebec City, QC Canada

**Keywords:** Pluripotent stem cells, Induced pluripotent stem cells, Cardiology, Cardiovascular diseases

## Abstract

Cardiomyocytes derived from patient-specific induced pluripotent stem cells (iPSC-CMs) successfully reproduce the mechanisms of several channelopathies. However, this approach involve cell reprogramming from somatic tissue biopsies or genomic editing in healthy iPSCs for every mutation found and to be investigated. We aim to knockout (KO) Na_V_1.5, the cardiac sodium channel, in a healthy human iPSC line, characterize the model and then, use it to express variants of Na_V_1.5. We develop a homozygous Na_V_1.5 KO iPSC line able to differentiate into cardiomyocytes with CRISPR/Cas9 tool. The Na_V_1.5 KO iPSC-CMs exhibited an organized contractile apparatus, spontaneous contractile activity, and electrophysiological recordings confirmed the major reduction in total Na^+^ currents. The action potentials (APs) exhibited a reduction in their amplitude and in their maximal rate of rise. Voltage optical mapping recordings revealed that the conduction velocity Ca^2+^ transient waves propagation velocities were slow. A wild-type (WT) Na_V_1.5 channel expressed by transient transfection in the KO iPSC-CMs restored Na^+^ channel expression and AP properties. The expression of Na_V_1.5/delQKP, a long QT type 3 (LQT3) variant, in the Na_V_1.5 KO iPSC-CMs showed that dysfunctional Na^+^ channels exhibited a persistent Na^+^ current and caused prolonged AP duration that led to arrhythmic events, characteristics of LQT3.

## Introduction

The human *SCN5A* gene located on chromosome 3p21 encodes the voltage-gated sodium channel (VGSC) α subunit, the predominant Na^+^ channel in the heart. Na_V_1.5 plays a vital role in triggering and shaping cardiac action potentials (APs). Its biophysical properties contribute to controlling phase zero of APs and their durations (APDs)^1^, and define the speed of propagation (conduction velocity) within the heart^2, 3^. Over the past 25 years, numerous *SCN5A* mutations have been associated with arrhythmic disorders, including congenital long QT syndrome type 3 (LQT3), Brugada syndrome (BrS), atrial fibrillation (AFib), progressive cardiac conduction defect (PCCD), sinus node dysfunction (SND), sudden infant death syndrome (SIDS), and dilated cardiomyopathy.

Most of the functional studies on specific *SCN5A* mutations have relied on heterologous expression systems. The first studies were performed in vitro using Xenopus oocytes^3^. Subsequently, cell lines such as human embryonic kidney (HEK293) cells and Chinese hamster ovary (CHO) cells were used. They provided a platform for characterizing the biophysical properties of Na^+^ channels and their variants. These heterologous expression systems remain in wide use today. The biophysical parameters of mutated Na^+^ channels obtained from electrophysiological recordings in these expression systems have been extrapolated to deduce their effect on cardiac function. The use of computational simulations to explore how Na_V_1.5 variants affect cardiac excitability by simulating AP firings has also proven useful for understanding their clinical impact. However, these expression and computational models have limitations. Although the Na_V_1.5 α subunit acts as a full-fledged Na^+^ channel in vivo, it is part of a large multiprotein complex. Cytoskeletal, regulatory, cell adhesion, trafficking, and gap junction proteins have been found in close association with Na_V_1.5, several of which alter the biological and/or biophysical properties of the channel.

Mouse models emerged as a tool for in vivo studies and a way to bypass the limitations of heterologous systems. The generation of transgenic mice carrying knockout channels or specific ion channel mutations has opened the way to studying the underlying disease mechanisms. However, mouse models are limited by the fundamental differences in cardiac electrophysiology between mice and humans and by the lack of general applicability of the results to human diseases^4^. In addition, the generation of such models is expensive, laborious, and far less straightforward than heterologous systems for studying and screening the functional effects of Na_V_1.5 variants.

Ideally, mutations associated with cardiac arrhythmias should be studied in the native cardiomyocyte environment. However, obtaining ventricular cardiac biopsies is a highly invasive procedure and is not without significant risk to the patient. Recent advances in induced pluripotent stem cells (iPSCs) have made it possible to generate an unlimited number of human cardiomyocytes (iPSC-CMs) in vitro from healthy individuals and from patients with cardiac abnormalities using specific differentiation protocols^5–8^. These advances have made it possible, among other things, to study the molecular determinants of channelopathies caused by mutated Na_V_1.5 channels in a human cardiac context^9^. When somatic tissues are unavailable, genomic editing with CRISPR/Cas9 tools, for example, can be used to introduce the mutation to be characterized into a control iPSC line and to then differentiate it into cardiomyocytes. Once again, this process of establishing an iPSC line for each Na_V_1.5 variant of interest is a laborious process and lacks the throughput of heterologous expression systems.

In the present study, we established an iPSC model that can be used to easily study various Na_V_1.5 channels variants in a human cardiac background. Our model is based on an iPSC line where the CRISPR/Cas9 genomic editing tool was used to permanently knock-out (KO) Na_V_1.5. We successfully differentiated this Na_V_1.5 KO cell line into cardiomyocytes and characterized the impact of the KO on cardiac function. We then used these cardiomyocytes (Na_V_1.5 KO iPSC-CMs) to express, by simple transfection, a mutated Na_V_1.5 channel (Na_V_1.5/delQKP) associated with LQT3 and to compare it with wild-type channel-transfected cells (Na_V_1.5/WT). The present study is a proof-of-concept for the use of Na_V_1.5 KO iPSC-CMs as an expression system to further characterize the impact of Na_V_1.5 variants in a human cardiac background.

## Methods

### iPSC cultures and cardiomyocyte differentiation

All the work with hiPSCs were approved by CIUSSS de la Capitale-Nationale ethic committee (Project #2019-1734). Human iPSCs were grown on hESC-qualified Matrigel (Corning, NY, USA) in mTeSRplus medium (StemCell Technologies, BC, Canada) and were routinely passaged with ReLeSR (StemCell Technologies). The iPSCs were produced at the LOEX core facility (Quebec, Qc, Canada) as described previously^10^. The iPSCs were differentiated into cardiomyocytes (CMs) with a monolayer-based protocol using STEMdiff™ cardiomyocyte differentiation kits (StemCell Technologies) according to the manufacturer's protocol and instructions. The differentiation process was very efficient, and spontaneously beating cells were observed at day 8 to 12 of differentiation. The iPSC-CM were maintained in STEMdiff™ Cardiomyocyte Maintenance media (StemCell Technologies) until the day of experiment.

### Generation of the NaV1.5 KO human iPSC line

The sgRNAs for CRISPR editing were designed using Predesigned Alt-R CRISPR-Cas9 guide RNA tool (Integrated DNA technologies website) and chosen according to the on-target and off-target scores. We have selected the following sgRNAs which have higher scores and the fewest and the non-cardiac off-targets: sgRNA-1 (5′-GATTTCAACCCTCCTAACAT) and sgRNA-2 (5′-ACTTGTAGCTGAGATCTGAG). The potential off-target sites are listed in the Supplementary Table S1 online.

**Figure 1:**
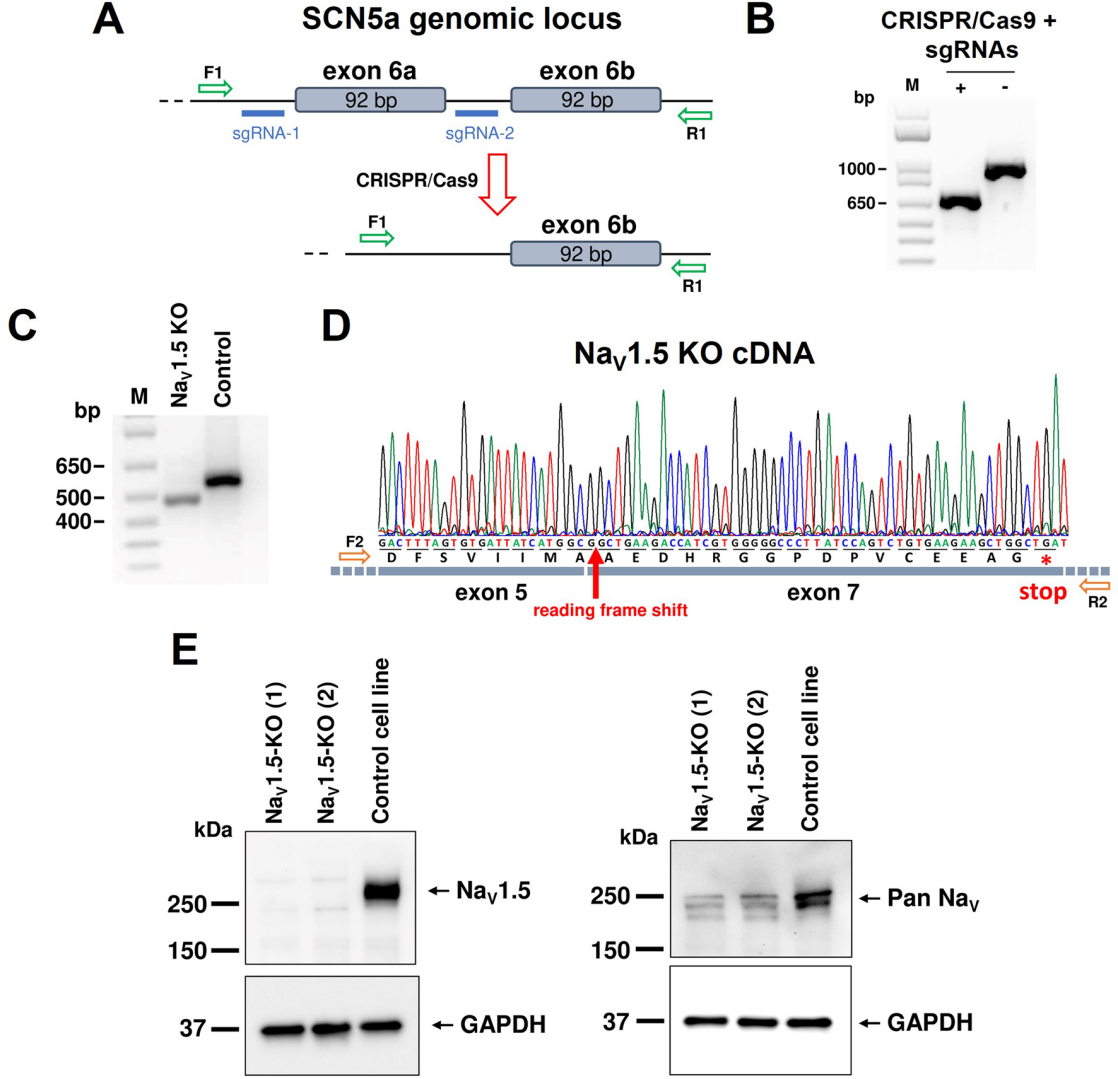
CRISPR-Cas9 genome editing to target *SCN5a*. (**A**) A schematic representation of the *SCN5a* locus and the sgRNA target sites located upstream and downstream from exon 6a. The sgRNA pair (blue) were designed to excise exon 6a in the *SCN5a* locus following CRISPR-Cas9 editing. The PCR primers used to screen and verify the nucleofected iPSC clones are shown in green. (**B**) PCR analysis of the positive iPSC clone used in this study amplified with primers shown in (**A**). Positive clone carrying a 685-bp PCR amplicon and non-nucleofected iPSC carrying a 962-bp amplicon. (**C**) PCR analysis on Na_V_1.5 cDNA using RNA extracted form iPSC-CM. The primers targeted the regions corresponding to exon 5 and exon 7 of Na_V_1.5 cDNA. PCR amplicons with a 92-bp deletion (Na_V_1.5 KO) or no deletion (control iPSC) corresponding to exon 6 are shown. (**D**) Sequencing chromatogram of the deleted PCR product in (**C**) from the Na_V_1.5 KO cell line showing the 92 bp deletion of exon 6 and the creation of stop codon induced by the frame shift of the reading frame. (**E**) Western blot analysis of Na_V_1.5 KO and control iPSC-CM. Anti-Na_V_1.5 (left panel) and the Anti-Pan Na_V_ antibodies (right panel) were used to assess the expression of Na^+^ channels. GAPDH staining was used as a loading control. Na_V_1.5 KO (1) and Na_V_1.5 KO (2) come from the same cell line but from two independent cardiac differentiation. This figure was made using MICROSOFT POWERPOINT Version 2106 (build 14131.20278 Office, https://docs.microsoft.com/en-us/) and IMAGEJ 1.52i (Java 1.8.0_66, http://imagej.nih.gov/ij).

The sgRNAs were cloned into the same modified px458 plasmid (Addgene plasmid 48,138) coding for the *S. aureus* Cas 9. Each sgRNA was under the control of its own U6 promoter. To generate CRISPR KO iPSCs, 7.5 µg of knockout plasmid (px458-sgRNA1-sgRNA2) was nucleofected into 800,000 iPSCs with 4D-Nucleofector (Lonza, Bâle, Switzerland) using P3 Primary cell kits (Lonza) and program CA-137. The cells were then seeded in 35-mm dishes in multiple dilutions in mTeSRplus supplemented with clone R (StemCell Technologies). After ~ 7 days in culture, each iPSC colony was manually picked using a micro-pipette under a Lynx EVO scope (Vision Engineering, CA, USA), and was transferred into two individual wells of two 96-well plates containing mTeSRplus medium. One plate used to extract the genomic DNA for PCR screening to detect positive clones, and the other was used to maintain and expand the positive clones. The PCR screening used the F1, 5′-GACCAGAAATGCACTTGCTTCCTGTA and R1, 5′-GGTGTCTATGAGAGTGGGCTTTGCT primers, which target regions surrounding the cutting sites of the sgRNAs. Positive clones, those with exon 6a removed, are characterized by a 682-bp PCR amplicon while negative clones are characterized by a 962-bp amplicon (Fig. 1B). One positive clone was chosen for the present study and was named Na_V_1.5 KO.

### cDNA analysis

RNA was extracted from Na_V_1.5 KO iPSC-CMs and the control cell line on day 30 of maturation using Quick RNA mini-prep kits (Zymo Research, CA, USA). cDNA was synthesized using the Protoscript II First Strand cDNA synthesis kit protocol (NEB, MA, USA). PCR analyses were performed using the F2, 5′-ACCTTCACCGCCATTTACA and R2, 5′-CGTGGTCGGGGTTCTCGCCTGC) primers, which target regions surrounding the locus corresponding to exon 6a or 6b on Na_V_1.5 mRNA. The PCR products were sent to a Sanger DNA sequencing facility (CHU de Québec, QC, Canada).

### Western blotting

The proteins of the iPSC-CMs were extracted on day 30 of maturation. Briefly, the cells were washed with PBS and were lysed by scraping the cells into lysis buffer (10 mM Tris, 1% Nonidet P-40, 0.5% deoxycholic acid) supplemented with protease inhibitor cocktail (MilliporeSigma, ON, Canada). The cells were incubated in the lysis buffer for 1 h at 4 °C. Then, they were clarified by centrifugation at 18,000 g for 15 min at 4 °C. Protein concentrations were measured using the DC protein assay (BioRad, ON, Canada), with BSA as a reference. Protein extracts (20 µg) were denatured in 2X Laemmli buffer (MilliporeSigma) at 37 °C for 30 min. They were resolved on 4–12% gradient stain-free SDS–polyacrylamide gels (Bio-Rad) and were blotted on 0.45-µm PVDF membranes (BioRad). The membranes were blocked and were incubated with rabbit anti-Na_V_1.5 (1:200, Cat #ASC-005, Alomone Labs, Israel) or anti-Pan Na_V_ antibodies (1:200, Cat #ASC-003, Alomone Labs, Israel) and rabbit anti-glyceraldehyde 3-phosphate dehydrogenase (GAPDH) (1:3000, Cat #2118, Cell Signaling Technology, USA). Horseradish peroxidase-conjugated anti-rabbit (1/3000, Cat #7074, Cell Signaling Technology) was used as secondary antibody. Proteins were revealed using the Clarity (Bio-Rad) Western ECL substrate and were visualized using a ChemiDoc system (Bio-Rad). The Western blots were performed on protein extracts from two independent Na_V_1.5 KO differentiations.

### Immunofluorescence staining

Immunofluorescence staining of iPSC-CMs was carried out on day 30 of maturation. The cells were dissociated from the monolayers using STEMdiff™ Cardiomyocyte Dissociation kits (StemCell Technologies) and were plated on Matrigel-treated 13-mm TC Coverslips (Sarstedt, QC, Canada). The cells were fixed in a mixture of 4% paraformaldehyde and 4% sucrose in PBS for 10 min. Then, they were washed and were permeabilized for 30 min at room temperature in a mixture of 0.1% Triton X-100, 1% BSA, and 5% goat serum in PBS. The cells were incubated overnight at 4 °C with the following antibodies: rabbit anti-MLC2v (1/300, Cat #ab89594, Abcam, ON, Canada) and mouse anti-cTnT (1/300, Cat #ab10214, Abcam). The cells were washed and were incubated for 1 h at room temperature with the appropriate Alexa secondary antibodies (1/250, Cat #A11005 and Cat #A11008, ThermoFisher Scientific, USA). DAPI was used to counterstain the nuclei. The cells were observed using a Zeiss LSM confocal microscope.

### iPSC-CM transfection

The iPSC-CMs were transfected at days 25–30 of maturation using Viafect™ Transfection Reagent according to an online protocol (Cat #E4981, Promega Corporation, WI, USA). The cells were transfected with the pCDNA3.1 vector containing either WT or delQKP Na_V_1.5 cDNA (500 ng) and with the pmGFP-N1 (250 ng) vector to select transfected cells based on green fluorescence signal. The medium was changed after 24 h, and the iPSC-CMs were dissociated into single cells in 35-mm dishes using STEMdiff™ Cardiomyocyte Dissociation kit (StemCell Technologies). Patch-clamp experiments were performed 3–7 days post-dissociation.

### Electrophysiology

Patch clamp experiments with the iPSC-CMs were performed at room temperature using an Axopatch 200B amplifier and pClamp software v10 (Molecular Devices, CA, USA). Macroscopic Na^+^ currents and APs were recorded using the whole-cell configuration of the patch clamp technique in voltage- and current-clamp modes, respectively. The pipettes were made from borosilicate glass capillaries (Sutter Instrument, CA, USA) and were fire polished.

For the voltage-clamp experiments, the pipettes were coated with HIPEC (Dow-Corning, MI, USA) to minimize electrode capacitance. The pipettes were filled with a solution containing (in mmol/L): 35 NaCl, 105 CsF, 10 EGTA, and 10 HEPES. The pH was adjusted to 7.4 with CsOH. To record biophysical parameters, the bath solution was composed of (in mmol/L): 105 NMDG, 35 NaCl, 2 KCl, 1.5 CaCl_2_, 1 MgCl_2_, 10 D-glucose, 10 HEPES, 10 TEA-Cl, and 0.01 Nifedipine. For persistent Na^+^ currents recordings, the bath solution was replaced by (in mmol/L): 140 NaCl, 2 KCl, 1.5 CaCl_2_, 1 MgCl_2_, 10 D-glucose, 10 HEPES, 10 TEA-Cl, 0.01 nifedipine, and 20 µmol/L TTX (LATOXAN, France). The pH of the bath solutions was adjusted to 7.4 with methanethiosulfonic (MTS) acid. Series resistance and cell capacitance were corrected. Na^+^ currents were filtered at 5 kHz, digitized at 10 kHz, and stored on a microcomputer equipped with an AD converter (Digidata 1440A, Molecular Devices). P/4 leak subtraction was used prior to applying pulse stimulations.

For the current-clamp experiments, the patch pipettes (resistance 2–5 mΩ) were filled with a solution containing (in mmol/L): 10 NaCl, 122 KCl, 1 MgCl_2_, 1 EGTA, and 10 HEPES. The pH was adjusted to 7.3 with KOH. The bath solution (external current clamp) was composed of (in mmol/L): 154 NaCl, 5.6 KCl, 2 CaCl_2_, 1 MgCl_2_, 8 D-glucose, and 10 HEPES. The pH was adjusted to 7.3 with NaOH. The APs were recorded at 0.5, 1, 2 and 2.5 Hz stimulation frequencies. The holding potential during recording was maintained at − 80 mV. The duration of stimulation pulse was 3 ms with 0.5–1.5 nA injected current depending on the cell.

### Optical mapping of iPSC-CMs in monolayers

Optical mapping techniques were used to study the membrane potential and calcium waves on iPSC-CMs monolayers. The iPSC-CMs monolayers were reconstituted with the same number of cells over the experiments. iPSC-CMs were dissociated at days 12 to 15 of maturation with STEMdiff™ Cardiomyocyte Dissociation kit (StemCell Technologies). The monolayer was then reconstituted with 350 000 cells seeded on a 13 mm TC coverslip (Sarstedt) coated with hESC-qualified Matrigel®. The STEMdiff™ Cardiomyocyte Maintenance medium was replaced every two days for at least 15 days post-reconstruction. The iPSC-CMs (30–60 days of maturation) were feed with fresh medium one hour before optical recording. The cells were stained for 15 min with 10 µmol/L of di-4-ANEPPS (Thermo Fisher Scientific) for membrane potentials recording or for 30 min with 5 µmol/L of Rhod-2-AM (Thermo Fisher Scientific) for intracellular Ca^2+^ transient imaging in the incubator (37 °C, 5% CO_2_). Both dyes were diluted in Tyrode solution (in mmol/L: 154 NaCl, 5.6 KCl, 2 CaCl_2_, 1 MgCl_2_, 8 D-glucose, and 10 HEPES; pH 7.3). The cells were washed with the Tyrode solution and incubated for an additional 10 min or 30 min before optical or Ca^2+^ imaging, respectively. Membrane voltage and intracellular Ca^2+^ transients were mapped at high-speed (500 frames/sec) using CMOS N256 camera (MiCAM03, Brainvision, SciMedia Ltd, U.S.A) at a field of view of 15 mm. The imaging system includes a 530 nm green LED light source (LEX2-LZ4-G) with a stable intensity of 360 mW/cm^2^, led by an optical fiber and followed by a imaging cube containing a collimator, a dichroic mirror (560 nm), a band-pass 531/40 nm excitation filter (50 mm, BrightLine®, Semrock) and a long-pass 665 nm emission filter (50 mm, Andover Corporation), as well as a lens system with a maximum aperture of f/1.4 aligned in epifluorescence configuration for di-4-ANEPPS and Rhod-2, AM imaging. The recordings were performed at 37 °C using TCW1 worming plate controlled by TC02 temperature controller (Multichannel Systems, BW, Germany). Bipolar platinum/iridium electrodes (World Precision Instruments) positioned at the lower edge of the preparation were used to pace the monolayer with a stimulus generator (STG4002, Multichannel Systems) at cycle of 500 ms to 1500 ms using a bipolar square pulse of ± 8 V of a duration of 10 ms. 10 µmol/L of blebbistatin (Sigma-Aldrich) was added to the recording solution during membrane voltage recording to prevent cardiomyocytes contraction artefacts in the fluorescence signal. Raw optical signals were processed using Brainvision Workbench software to analyse the electrophysiological measurements, calculate the conduction velocities and generate the maps.

### Statistical analysis

In patch clamp recordings, APs exhibited diverse shapes that can be attributed to different cardiomyocyte cell types, that are, ventricular, atrial, and nodal cells. For statistical analyses, we decided to pool ventricular and atrial-like APs and exclude nodal-like APs. The nodal-like APs were excluded based on their very distinctive shape, i.e., no plateau and low overshoot.

All statistical analyses were performed using PRISM8 software (GraphPad, CA, USA). The normality of distribution was determined using D’Agostino-Pearson normality test. When the distribution is normal, the data are expressed as mean ± SEM (standard error of the mean). If not, the data are expressed as median ± quartiles (25 % and 75 %) with the min to max values. When 3 groups were compared, statistical significance was determined by one-way ANOVA with Dunnet’s post hoc test, or a two-way ANOVA for two independent variables. When only two groups were compared, a two-tailed unpaired Student’s t-test was used. Where data were not normally distributed, a nonparametric test (Mann–Whitney or Kruskal–Wallis test) was performed. All the statistical tests were performed using a 95 % confidence interval and the differences were considered significant beyond the risk threshold 0.05% (*P < 0.05, **P < 0.01, ***P < 0.001).

## Results

### CRISPR-Cas9 strategy for Na_V_1.5 knockout

To selectively and permanently suppress Na_V_1.5 channel expression in iPSCs, we used CRISPR-Cas9 gene editing technology. The strategy was to remove exon 6a in the *SCN5a* gene using two sgRNAs that precisely target the regions flanking this exon (Fig. 1A). We used PCR amplifications of the *SCN5a* genomic locus encompassing the sgRNA target sites to screen for positive iPSC clones. Figure 1B shows the positive PCR result of the Na_V_1.5 KO cell line used in the present study. The Na_V_1.5 KO cells had a shorter amplicon than the control cells (not exposed to Cas9), confirming that exon 6a in *SCN5a* had been deleted. A genetic screening by quantitative PCR was performed of the Na_V_1.5 KO iPSCs to verify the chromosomes status (Supplementary Fig. S1 online). 8 most mutated regions in iPSCs were analysed and compared to a genomic DNA control provided in the hPSC Genetic Analysis Kit. The NaV1.5 KO iPSCs has the same copy number that control except for the X chromosome where it was divided by two. The NaV1.5 KO cell line comes from a male, containing one copy of X chromosome, while the genomic DNA control comes from a female. So, the KO line does not appear to have genetic abnormalities.

To verify that Na_V_1.5 protein expression was suppressed in the cells, the Na_V_1.5 KO iPSCs were differentiated into cardiomyocytes. After 30 days of maturation, RNA and Western blot analyses were performed. The Na_V_1.5 cDNA PCR amplification revealed a shorter amplicon, confirming that the Na_V_1.5 mRNA was free of exon 6a and 6b as shown by the sequencing chromatogram (Fig. 1C,D). The deletion of the two exons 6 in the mRNA instead of only one as designed by our strategy can probably be explained by a mis-splicing event. The deletion of the exon 6a may have compromised splicing sites for the exon 6b since they are only 140 bp apart in the SCN5a gene. The Na_V_1.5 cDNA sequencing chromatogram validated the shift in the reading frame of Na_V_1.5 and the formation of stop codons in the region corresponding to exon 7 (only the first stop codon is shown in Fig. 1 D). The Western blot analysis were performed using two primary antibodies (Fig. 1E). The anti-Na_V_1.5 antibody which recognize the intracellular loop of Na_V_1.5 channel, between domains I and II and the anti-Pan Na_V_ antibody which recognize the intracellular loop between domains III and IV, identical in all isoforms of Na_V_1 in all vertebrates. The unedited blots are available at different exposures in Supplementary Figure S6 and S7. The immunostaining shows that Na_V_1.5 protein expression was completely suppressed in the KO iPSC-CMs. Our CRISPR construction makes it possible to stop the translation of the protein at the end of domain I, that is why we cannot observe a truncated Na_V_1.5 protein in the KO iPSC-CMs. The immunostaining with an anti-Pan Na_V_ antibody that recognizes all channel subtypes showed that isoforms other than Na_V_1.5 were expressed but at much lower levels (Fig. 1E). We used this new Na_V_1.5 KO iPSC line in the present study.

### Intact contractile structures in Na_V_1.5 KO iPSC-CMs

**Figure 2:**
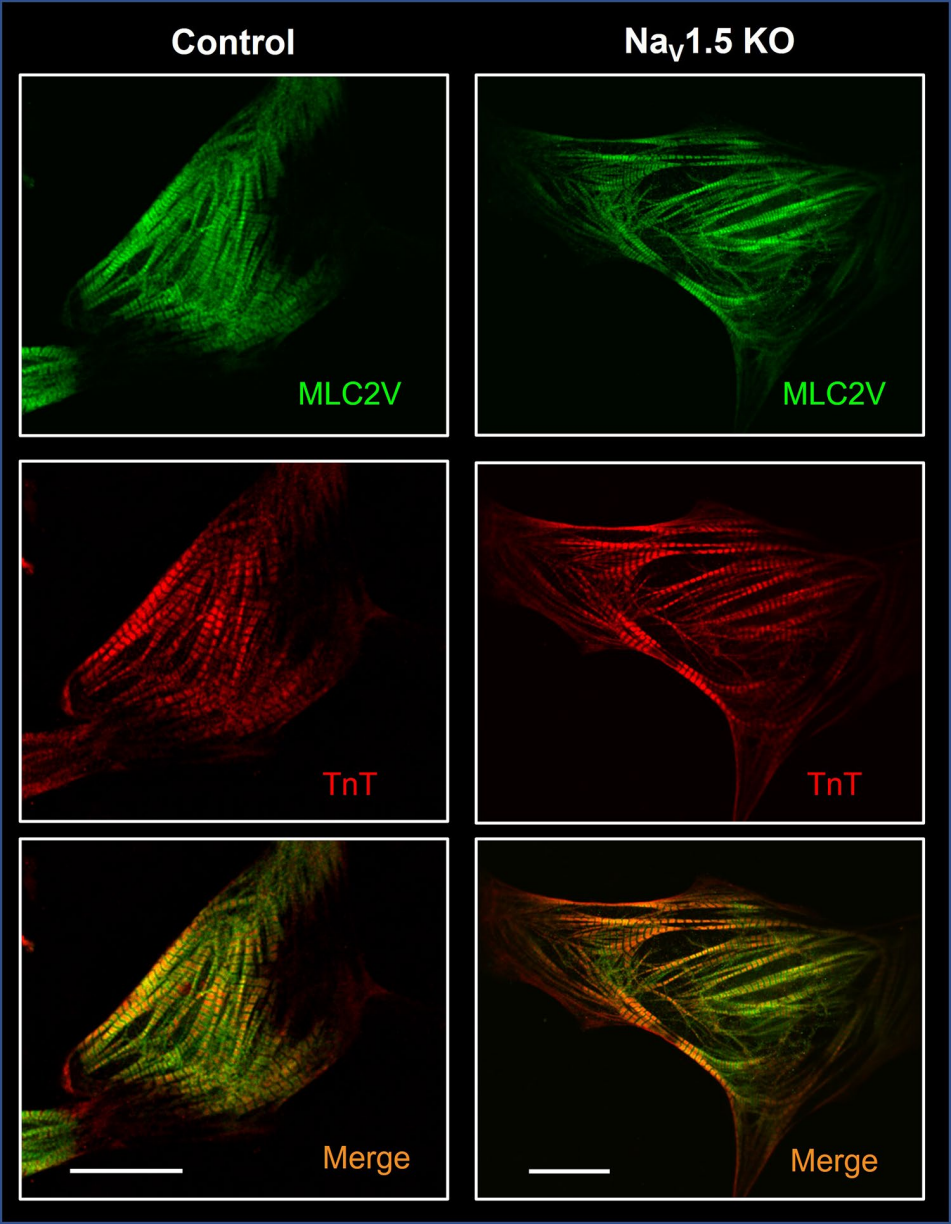
Immunofluorescence staining of cardiac markers in Na_V_1.5 KO iPSC-CMs. NaV1.5 KO iPSC-CMs with organized contractile proteins, including myosin light chain 2v (MLC2v, green staining) and cardiac troponin T (cTnT, red staining). The bottom panels show merged images. Scale bar: 20 µm. Images were acquired using a Zeiss ImagesM2 LSM confocal microscope and were processed using ZEN software (Zeiss). This figure was made using IMAGEJ 1.52i (Java 1.8.0_66, http://imagej.nih.gov/ij).

The Na_V_1.5 KO iPSCs were successfully differentiated into cardiomyocytes (Supplementary Videos S1 and S2 online). No difference in the differentiation course of the KO cells was observed compared to the control cells. The Na_V_1.5 KO iPSC-CMs started to spontaneously beat around day 8 to 10 of differentiation as did the control cells. After 30 days of maturation, the beating rate of the Na_V_1.5 KO iPSC-CMs (0.46 ± 0.30 Hz) was also similar to that of the control cells (0.46 ± 0.19 Hz) (Fig. 5I). The morphologies of the cardiomyocytes differentiated from the Na_V_1.5 KO iPSC line were then characterized to rule out the possibility that the loss of Na_V_1.5 had affected sarcomeric organization. Immunofluorescence staining for myosin light chain 2v (MLC2v) and cardiac troponin T (cTnT) revealed that the contractile proteins in Na_V_1.5 KO iPSC-CMs exhibited the same normal organization as in the control cells (Fig. 2).

### Voltage and current-clamp analyses of Na_V_1.5 KO iPSC-CMs

**Figure 3:**
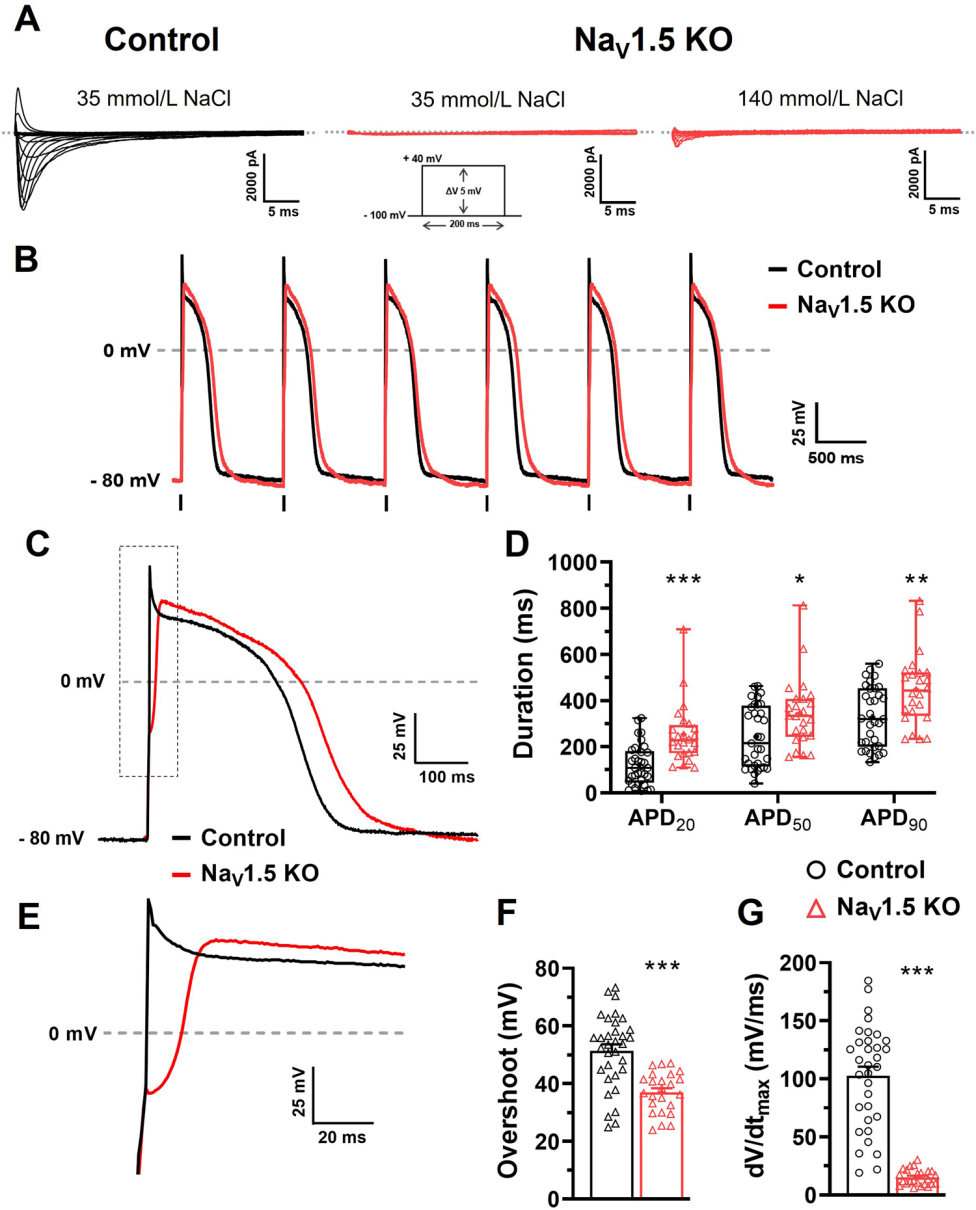
Electrophysiological characterization of Na_V_1.5 KO iPSC-CMs. (**A**) Representative Na^+^ currents recorded in control and Na_V_1.5 KO iPSC-CMs with 35 or 140 mM NaCl in the external solution. The dashed line represents zero current. The currents were obtained using 200 ms pulses from − 100 to + 40 mV in + 5 mV increment. (**B**) Superposed train of APs from control and Na_V_1.5 KO at 1 Hz. Vertical bars at the bottom of the traces indicate the 3 ms stimulation pulse. (**C**) Close-up view of one AP showing the increase in the APD for Na_V_1.5 KO. (**D**) Box and whiskers summarizing the median (with quartiles and min to max values) of APD at 20 %, 50 %, or 90 % of repolarization at 1 Hz. *p < 0.05, **p < 0.01, ***p < 0.001 as determined using a nonparametric Mann–Whitney test. (**E**) Close-up view on the rising phase of representative APs traces. (**F,G**) Bar graphs showing the mean ± SEM of overshoot (**F**) and the maximal upstroke velocity (dV/dt_max_) (**G**) of APs recorded at 1 Hz. ***p < 0.001 as determined using an unpaired t-test. Control n = 33 versus Na_V_1.5 KO n = 24. This figure was made using MICROSOFT POWERPOINT Version 2106 (build 14131.20278 Office, https://docs.microsoft.com/en-us/) and PRISM 8 Version 8.0.2 (263, https://www.graphpad.com/).

The Na_V_1.5 channel is by far the main contributor to Na^+^ currents that initiate and trigger the rise of APs in cardiomyocytes. With the KO of Na_V_1.5, we expected that the iPSC-CMs would produce a very small Na^+^ current. We thus performed voltage-clamp recordings in dissociated iPSC-CMs cells to measure the Na^+^ and Ca^2+^ currents in the Na_V_1.5 KO and control cells. The Na_V_1.5 KO iPSC-CMs did not produce any recordable Na^+^ current, whereas the control cells generated a mean current amplitude of − 3200 pA, as shown by the representative current traces (Fig. 3A). When the Na^+^ concentration was increased from 35 to 140 mmol/L in the external recording solution, the Na_V_1.5 KO iPSC-CMs produced a small Na^+^ current of –670 pA, as shown by the representative current trace (Fig. 3A). At this Na^+^ concentration, the amplitudes of the Na^+^ currents in the control cells were too large (> − 20,000 pA) to be recorded. Given that the Na_V_1.5 KO cell line generated beating cardiomyocytes, it should also produce Ca^2+^ currents that are responsible for excitation–contraction coupling in cardiac muscles. The Na_V_1.5 KO produce a Ca^2+^ current which has the same biophysical properties that control iPSC-CMs (Supplementary Fig. S2 online).

In the Na_V_1.5 KO iPSC-CMs, the small residual Na^+^ current was blocked by 50 nmol/L TTX (Supplementary Fig. S3 online). The Ca^2+^ current activated several milliseconds after the Na^+^ current was blocked with 5 µmol/L nifedipine, a Ca^2+^ channel blocker.

We next studied the impact of Na_V_1.5 current losses on the properties of APs in iPSC-CMs. Na_V_1.5 KO and control iPSC-CMs both triggered spontaneous APs in the gap-free mode of the current-clamp configuration (data not shown). We observed similar cell-subtypes repartition between control and Na_V_1.5 KO iPSC-CMs: most ventricular-like cells and a minority of atrial- and nodal-like cells (data not shown). When stimulated at a stimulation frequency of 1 Hz, the Na_V_1.5 KO cells triggered AP trains that respected the pace of stimulation seen in the control cells (Fig. 3B). However, the injected current to trigger an AP in the Na_V_1.5 KO cells (0.97 ± 0.05 nA) was significantly increased compared to control cells (0.77 ± 0.04 nA; **p < 0.01 unpaired t-test). The following experiments were carried out on cardiomyocytes stimulated at 1 Hz in order to normalize the beating rate and enable further analysis. For each AP, several parameters were analysed, this include the overshoot, the dV/dt_max_ which corresponds to the opening of Na^+^ channels (0 phase of AP), and the 20, 50 and 90% of repolarization which corresponds to the opening of Ca^+^ and K^+^ channels (1, 2 and 3 phases of AP).

As expected, several alterations in the APs from Na_V_1.5 KO cells were identified. The APs from the Na_V_1.5 KO iPSC-CMs exhibited a significant increase in duration (APD) when measured at 20, 50, and 90% of repolarization compared to the control cells (Fig. 3C,D). For example, the median of APD_90_ was 442.5 ms with quartiles [333.9; 522.0] for the Na_V_1.5 KO cells compared to 319.3 ms [200.3; 452.8] for the control cells, which was a significant increase of 122 ms. The AP overshoot and the upstroke velocity (dV/dt_max_) were also affected in the Na_V_1.5 KO iPSC-CMs, which influenced the amplitude and the time to peak of APs, as shown in the representative traces (Fig. 3E). The overshoot in the Na_V_1.5 KO iPSC-CMs (37.0 ± 1.4 mV) decreased significantly by 14 mV compared to the control cells (51.4 ± 2.2 mV) (Fig. 3F). The main effect on APs was related to the dV/dt_max_, where a major decrease of 88 mV/ms was observed in the Na_V_1.5 KO iPSC-CMs (15.2 ± 1.4 mV/ms) compared to the control cells (102.8 ± 7.7 mV/ms) (Fig. 3G).

From a pharmacological point of view, Na_V_1.5 KO does not respond in the same way as the control iPSC-CMs. The APs from control iPSC-CMs were sensitive to TTX treatment (Supplementary Fig. S4 online). Indeed, the addition of TTX decreased the dV/dt_max_ of APs from control cells and this in a concentration-dependent way. For Na_V_1.5 KO iPSC-CMs, the TTX had no blocking effect of the triggering of APs (Supplementary Fig. S3 online). Moreover, the treatment with 1 µmol/L nifedipine blocks the APs from the Na_V_1.5 KO but not APs from the control iPSC-CMs which just had a shortened duration (Supplementary Fig. S3 and S5 online). These results confirm the Ca^2+^ nature of the APs recorded from Na_V_1.5 KO iPSC-CMs.

### Voltage and calcium optical mapping of iPSC-CM monolayers

**Figure 4:**
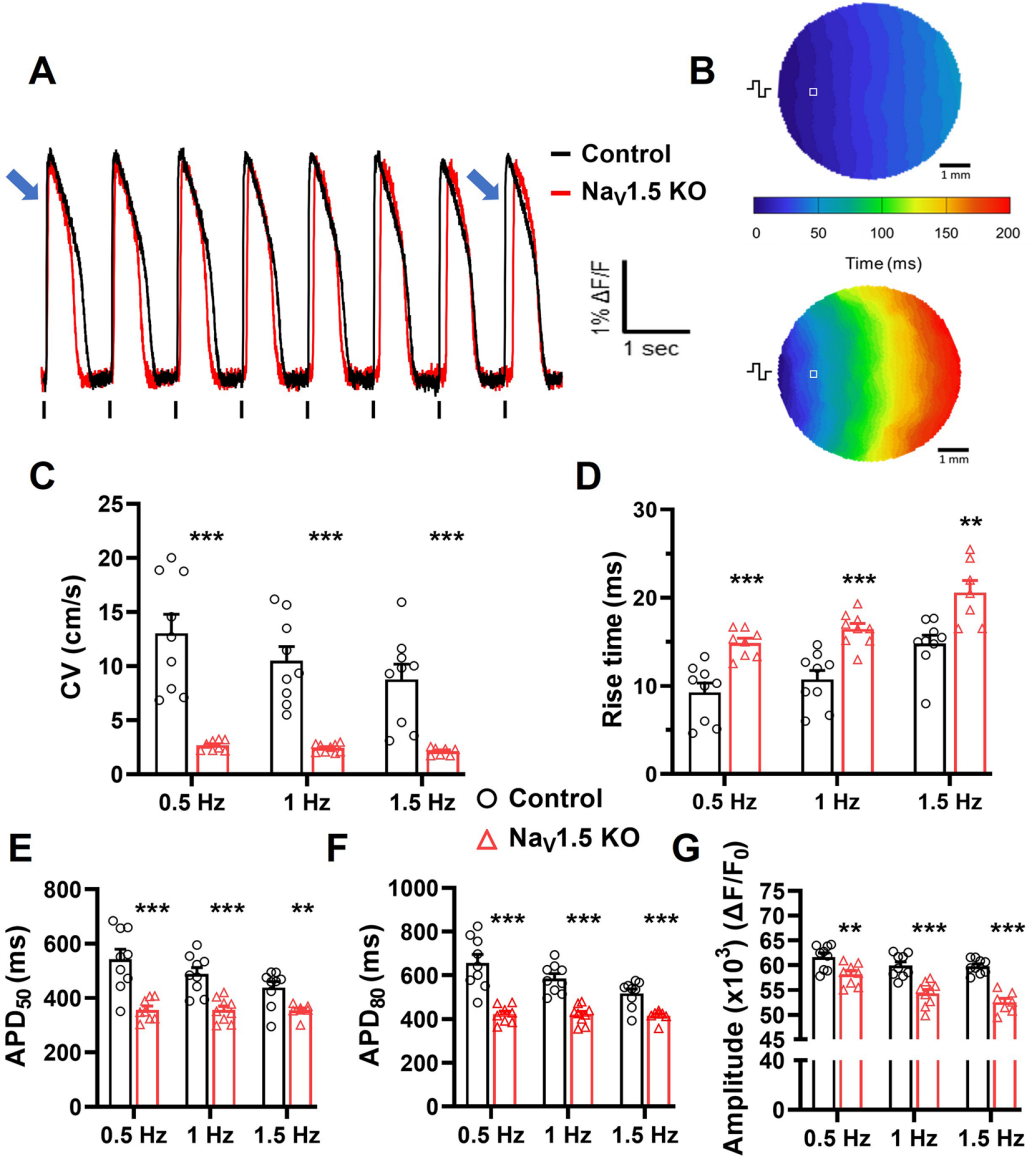
Slower conduction velocity and altered OAP parameters in Na_V_1.5 KO iPSC-CMs. (**A**) Representative optical action potentials (OAPs) recorded in control and Na_V_1.5 KO iPSC-CMs monolayers using di-4-ANEPPS dye. Blue arrows at the beginning and at the end of the pulse train indicate the cumulative delay of OAPs activation in Na_V_1.5 KO recording. (**B**) Representative activation maps at a pacing of 1 Hz. The  symbol indicating the position of stimulating electrodes, and **□** showing the position of the representative recordings. (**C**) Bar graphs summarizing the CVs, (**D**) the rise time, (**E,F**) action potential durations at 50 % or 80 % of repolarization and (**G**) the normalized amplitude of OAPs measured at stimulation frequencies of 0.5, 1 and 1.5 Hz in control (n = 9) and Na_V_1.5 KO (n = 7**–**9) iPSC-CM monolayers. Bars indicate SEM. **p < 0.01, ***p < 0.001 (control vs. Na_V_1.5 KO) as determined using unpaired t-test. A replicate (n) represents a single monolayer of iPSC-CMs on which optical mapping was performed. This figure was made using MICROSOFT POWERPOINT Version 2106 (build 14131.20278 Office, https://docs.microsoft.com/en-us/) and PRISM 8 Version 8.0.2 (263, https://www.graphpad.com/).

We next generated large (~ 1 cm diameter) iPSC-CM monolayers to study the biophysical impact of the loss of Na_V_1.5 channels at level closer to heart tissue using the optical mapping (OM) technique. The conduction of electrical impulses through the iPSC-CM monolayers stained with the voltage sensitive dye di-4-ANEPPS was first analyzed. Figure 4A shows representative optical APs (OAPs) recorded in Na_V_1.5 KO and control monolayers stimulated at 1 Hz. As with the patch-clamp recordings, both cell lines generated AP trains that followed the pace of the electrical stimulations. Activation maps were produced for the Na_V_1.5 KO and control monolayers from these optical recordings (Fig. 4B). The maps notably showed that the conduction velocity (CV) was slower in the Na_V_1.5 KO monolayers than in the control monolayers (Supplementary Video S3 online). When stimulated at 0.5, 1, and 1.5 Hz, the conduction velocities measured in the Na_V_1.5 KO monolayers were 2.67 ± 0.16, 2.43 ± 0.13, and 2.12 ± 0.13 cm/s, respectively compared to 13.02 ± 1.7, 10.49 ± 1.3, and 8.77 ± 1.4 cm/s in the control monolayers, respectively (Fig. 4C). We next analyzed the parameters of the OAPs and found that the rise time, the duration, and the amplitude were affected in the Na_V_1.5 KO monolayers at all three stimulation frequencies (Fig. 4D). The rise time of the OAPs was prolonged in the Na_V_1.5 KO monolayers by an average of 5.7 ms. For example, the rise time was 16.5 ± 0.6 and 10.7 ± 1.0 ms for the Na_V_1.5 KO and the control monolayers at 1 Hz, respectively. The duration of the OAPs was significantly shortened for the Na_V_1.5 KO monolayers (Fig. 4E,F; Supplementary Video S3 online). At 1 Hz, the APD_50_ and APD_80_ were 356 ± 15 and 421 ± 14 ms for the Na_V_1.5 KO monolayers, and 489 ± 24 and 585 ± 22 ms for the control monolayers. This represented 133 ms and 164 ms differences in the APD_50_ and APD_80_, respectively. The amplitude of the OAPs also significantly decreased by 9 %, as calculated using the normalized fluorescence intensity, decreasing from 59,900 relative fluorescence units (RFU) in the control monolayers to 54,275 RFU in the Na_V_1.5 KO monolayers at 1 Hz (Fig. 4G).

**Figure 5:**
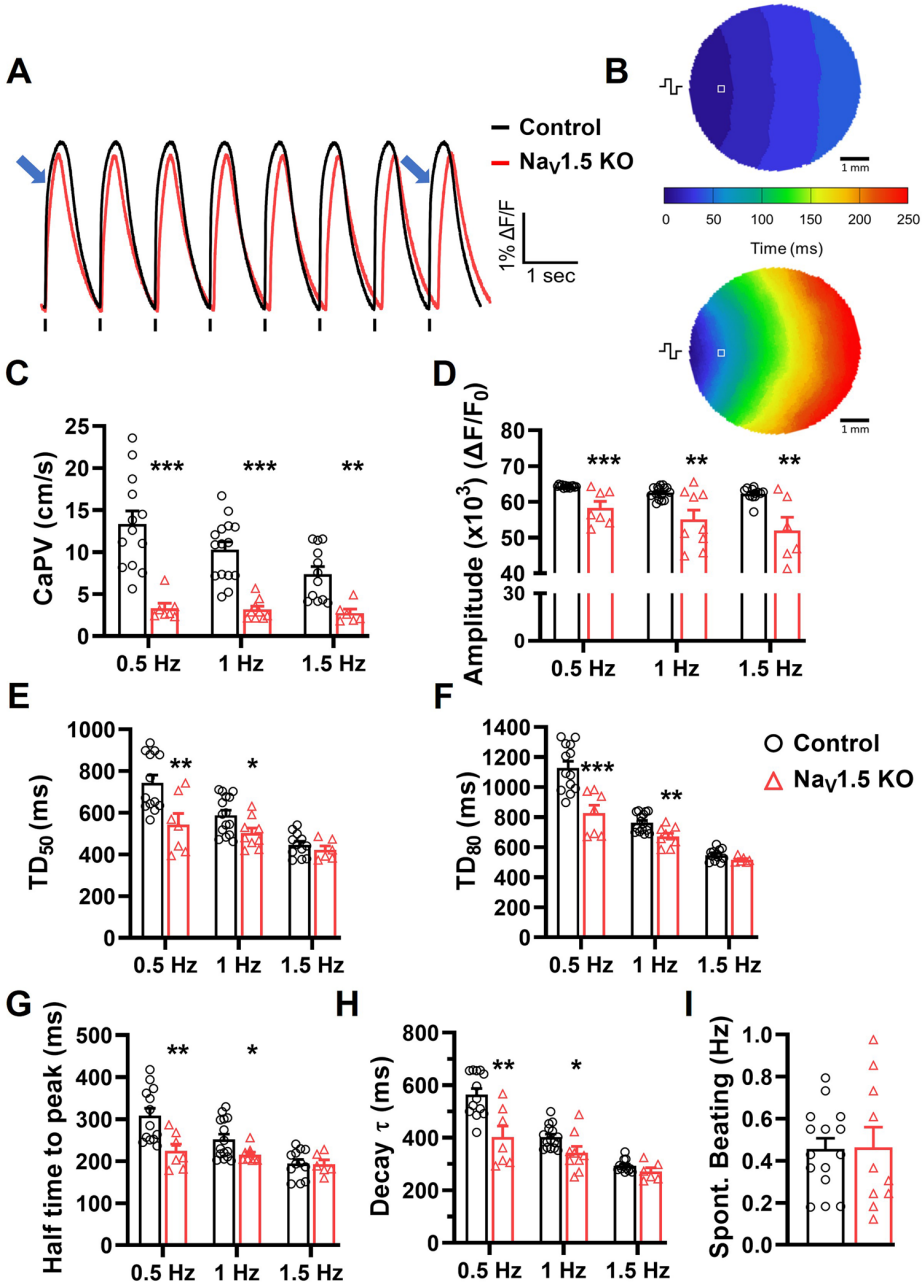
Altered calcium handling in Na_V_1.5 KO iPSC-CMs monolayers. (**A**) Representative Ca^2+^ transient traces of control and Na_V_1.5 KO iPSC-CMs monolayers. Blue arrows indicate the Ca^2+^ activation delay in Na_V_1.5 KO recording. (**B**) Representative  Ca^2+^ activation maps at pacing of 1 Hz. The symbol indicating the position of stimulating electrodes, and **□** showing the position of the representative recordings. (**C**) Bar graphs summarizing the Ca^2+^ propagation velocities (CaPVs), (**D**) the normalized amplitude. (**E,F**) the Ca^2+^ transient durations at 50 and 80% of the repolarization phase (TD_50_ and TD_80_), (**G**) the half time to peak, and (**H**) the time constant of repolarization (Decay tau) measured at stimulation frequencies of 0.5, 1 and 1.5 Hz in control (n = 12**–**15) and Na_V_1.5 KO (n = 6**–**9). (**I**) Bar graphs showing the spontaneous Ca^2+^ waves frequencies in control (n = 15) and Na_V_1.5 KO (n = 10) iPSC-CM monolayers. A replicate (n) represents a single monolayer of iPSC-CMs on which the optical mapping was performed. Bars indicate SEM. *p < 0.05, **p < 0.001, ***p < 0.001 as determined by unpaired t-test. This figure was made using MICROSOFT POWERPOINT Version 2106 (build 14131.20278 Office, https://docs.microsoft.com/en-us/) and PRISM 8 Version 8.0.2 (263, https://www.graphpad.com/).

The spatial changes in the kinetics and amplitude of Ca^2+^ transients were next studied using the fluorescent probe Rhod-2-AM on cell monolayers. Figure 5A shows representative Ca^2+^ signal recorded in Na_V_1.5 KO and control monolayers when electrically stimulated at 1 Hz. Uniform propagation of Ca^2+^ waves through the entire monolayer was observed in both cell lines, as shown by the representative Ca^2+^ activation maps in Fig. 5B. As observed in OAPs recording, Na_V_1.5 KO monolayers exhibited a significantly delayed and slower propagation of Ca^2+^ transients and a compared to control monolayers (Supplementary Video S4 online). This was expected since the Ca^2+^ transients are triggered during AP propagation through the Ca^2+^-induced Ca^2+^ release (CICR) process. Thus, Ca^2+^ waves propagation velocities (CaPV) were measured in Na_V_1.5 KO (3.3 ± 0.6, 3.2 ± 0.4 and 2.7 ± 0.5 cm/s) and control (13.4 ± 1.5, 10.3 ± 0.93 and 7.4 ± 0.9 cm/s) at 0.5, 1 and 1.5 Hz pacing frequencies, respectively (Fig. 5C). Similarly, the Ca^2+^ transient characteristics in Na_V_1.5 KO monolayers were altered including its amplitude, durations (TD_50,80_), half times to peak and decay speed (τ). A significant reduction of 10% in the Ca^2+^ transients normalized amplitudes was observed between the two groups reflecting the maximum intracellular Ca^2+^ concentrations, as measured at 64,335 RFU in the control and 58,337 RFU in the Na_V_1.5 KO monolayers at stimulation frequency of 1 Hz (Fig. 5D). Furthermore, the durations of Ca^2+^ transients were significantly shortened in Na_V_1.5 KO iPSC-CMs at 0.5 Hz and 1 Hz. For example, TD_50_ and TD_80_ were reduced by 85 and 91 ms at 1 Hz, respectively (503 ± 24.4 and 672.8 ± 21.2 ms for Na_V_1.5 KO, compared to 588.6 ± 23.8 and 763.4 ± 15.9 ms for control) (Fig. 5E,F). More, the half times of Ca^2+^ peak was shortened in Na_V_1.5 KO iPSC-CMs at 0.5 Hz and 1 Hz, but not at 1.5 Hz (Fig. 5G). At 1 Hz, the half time of Ca^2+^ peak was decreases by 36 ms with 252.3 ± 12.1 and 216 ± 5.8 ms for control and Na_V_1.5 KO, respectively. As for the decay tau (τ), Na_V_1.5 KO iPSC-CMs exhibit a faster decay of Ca^2+^ transients (smaller τ) at 0.5 Hz and 1 Hz (Fig. 5H; Supplementary Video S4 online). As an example, the decay τ was 342.6 ± 24.5 in Na_V_1.5 KO, and 402.5 ± 11.5 in control at 1 Hz. Finally, the spontaneous beating frequencies were measured before the addition of blebbistatin and electrical stimulation for each monolayer. The beating frequencies were found similar at ~ 0.46 Hz within the two groups (Fig. 5I).

### Na_V_1.5 channel re-expression in Na_V_1.5 KO iPSC-CMs

**Figure 6:**
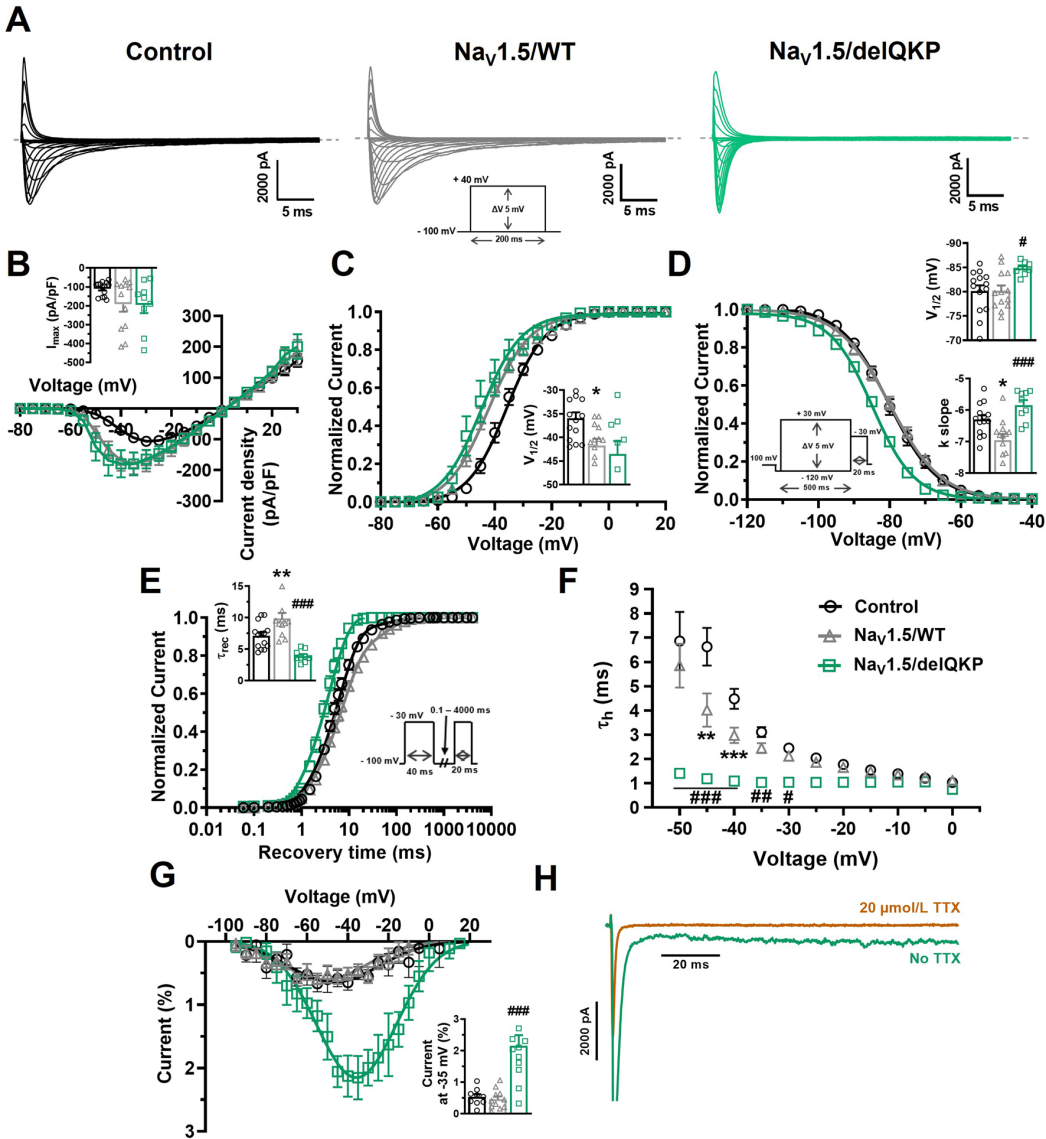
Altered biophysical properties of Na^+^ channel in KO iPSC-CMs expressing Na_V_1.5/ΔQKP. (**A**) Representative Na^+^ currents recorded in control and KO iPSC-CMs transfected with Na_V_1.5/WT or Na_V_1.5/delQKP. The dashed line represents zero current. The currents were obtained using 200 ms pulses from − 100 to + 40 mV in + 5 mV increment. (**B**) Normalized intensity/voltage relationships (I/V) recorded in control (n = 14), Na_V_1.5/WT (n = 13) and Na_V_1.5/delQKP (n = 9) iPSC-CMs. Na^+^ current densities were measured by normalizing current amplitudes to membrane capacitance. Inset shows graph of I_max_. (**C**) Steady-state activation of Na^+^ currents. Activation curves were generated using a standard Boltzmann distribution: G(V)/G_max_ = 1/(1 + exp (− (V − V_1/2_)/k)). Inset shows graph of V_1/2_. (**D**) Steady-state inactivation of control (n = 14), Na_V_1.5/WT (n = 13) and Na_V_1.5/delQKP (n = 9). Inactivation currents were obtained using 20-ms test pulses to − 30 mV after a 500-ms pre-pulse to potentials ranging from − 120 to + 30 mV. The inactivation values were fitted to a standard Boltzmann equation: I(V)/I_max_ = 1/(1 + exp ((V − V_1/2_)/k)) + C. Inset shows graph of V_1/2_ and k slope (**E**) Recovery from inactivation values recorded from control (n = 14), Na_V_1.5/WT (n = 12) and Na_V_1.5/delQKP (n = 9) iPSC-CMs. The cells were depolarized to − 30 mV for 40 ms from a holding potential of − 100 mV to inactivate the Na^+^ channels. Test pulses were then applied at − 30 mV for 20 ms to measure current amplitudes, with an interval ranging from 0.1 to 4000 ms. The resulting curves were fitted with a simple exponential equation: (A (exp (− t/τ) + C). Inset shows a graph of the recovery time constant (τ_rec_). (**F**) The time constants of fast inactivation decay were plotted as a function of voltage for control (n = 14), Na_V_1.5/WT (n = 13) and Na_V_1.5/delQKP (n = 9). The time constants were obtained using a simple exponential function. (**G**) Persistent Na^+^ current/voltage relationships recorded in control (n = 9), Na_V_1.5/WT (n = 11) and Na_V_1.5/delQKP (n = 12) iPSC-CMs. The currents were obtained using 300 ms pulses from − 100 to + 40 mV in + 5 mV increment. Persistent Na^+^ currents are represented as percentage of the current, obtained by normalizing the values at the end of the pulse to the maximum transient current. Inset shows graph of percentage of current at − 35 mV. (**H**) Representative persistent Na^+^ current at − 35 mV recorded in Na_V_1.5/delQKP iPSC-CMs before and after adding 20 µmol/L TTX. Bars indicate SEM. *p < 0.05, **p < 0.01 (control vs. Na_V_1.5/WT) and #*p* < 0.05, ##*p* < 0.01, ###*p* > 0.001 (Na_V_1.5/WT vs. Na_V_1.5/ΔQKP) using ANOVA and Dunnett’s post hoc tests. This figure was made using MICROSOFT POWERPOINT Version 2106 (build 14131.20278 Office, https://docs.microsoft.com/en-us/) and PRISM 8 Version 8.0.2 (263, https://www.graphpad.com/).

We next used the Na_V_1.5 KO cell line as an expression system to characterize the impact of a Na_V_1.5 variant in a human cardiac environment. The known LQT3 variant delQKP_1507-1509_ (delQKP) was used as a proof-of-concept in the present study. The Na_V_1.5 KO iPSC-CMs were transiently transfected with either the cDNA Na_V_1.5/delQKP construct or the Na_V_1.5/WT construct. We conducted a voltage-clamp analysis and compared the biophysical properties of the mutated channel with those of the WT channel. The Na_V_1.5/WT iPSC-CMs were also compared with the control iPSC-CMs. The Na_V_1.5 KO iPSC-CMs transfected with Na_V_1.5/WT (Na_V_1.5/WT) or Na_V_1.5/delQKP both produced a robust typical Na^+^ current (Fig. 6A). The current densities in Na_V_1.5/WT (− 192 ± 39 pA/pF) and Na_V_1.5/delQKP (− 195 ± 43 pA) were similar as shown by the I/V curves (Fig. 6B). The Na_V_1.5/WT tended to have slightly higher current densities compared to control iPSC-CMs (109 ± 10 pA/pF) but the difference was not significant (*p* = 0521) (Fig. 6B). The I/V curves were converted to conductance-voltage curves (G/V), which revealed no significative differences in steady-state activation between Na_V_1.5/WT and Na_V_1.5/delQKP (Fig. 6C). Compared to the control iPSC-CMs (− 35.9 ± 1.2 mV), the V_1/2_ of activation of Na_V_1.5/WT (− 41.7 ± 1.4 mV) was shifted significantly toward more negative voltages (Fig. 6C). An analysis of steady-state inactivation revealed a significant − 4.6 mV shift in the V_1/2_ of Na_V_1.5/delQKP (− 84.8 ± 0.4 mV) compared to Na_V_1.5/WT (− 80.2 ± 1.1 mV) (Fig. 6D). No difference was seen in the V_1/2_ of inactivation between Na_V_1.5/WT and the control iPSC-CMs. The voltage dependence of inactivation was also significantly higher in Na_V_1.5/delQKP (k_v_ = − 5.8 ± 0.2) than in Na_V_1.5/WT (k_v_ = − 7.0 ± 0.2) as shown by the slope of the inactivation curve (Fig. 6D). We next used a double-pulse protocol to directly measure the kinetics of recovery from inactivation. The recovery kinetics, which were determined by fitting a single-exponential equation, were more than two times faster for Na_V_1.5/delQKP (t_rec_ = 3.9 ± 0.3 ms) than for Na_V_1.5/WT (t_rec_ = 9.9 ± 0.9 ms) (Fig. 6E). Na_V_1.5/WT was slightly slower to recover than the control (t_rec_ = 7.1 ± 0.5 ms). The decay of inactivation of the mutated channel was also affected. Na_V_1.5/delQKP exhibited three times faster inactivation than Na_V_1.5/WT as calculated by fitting the current decays with an exponential function (Fig. 6F). For example, at − 40 mV, the time constants of decay were 1.1 ± 0.1 ms and 3.0 ± 0.3 ms for Na_V_1.5/WT and Na_V_1.5/delQKP, respectively. This faster inactivation rate can be clearly seen on the representative current traces in Fig. 6A. These results indicate that the inactivation process of the channels was compromised by the deletion of the three amino acids. This situation often generates the appearance of a persistent Na^+^ current, which is what occurred with the variant channel. Na_V_1.5/delQKP produced a persistent Na^+^ current more than four times larger than Na_V_1.5/WT, reaching 2.2 ± 0.3%, while it was at 0.5 ± 0.2% of the transient current in Na_V_1.5/WT (Fig. 6G). The nature of this non-inactivating current was confirmed by its block with 20 µmol/L TTX (Fig. 6H). The biophysical parameters of the voltage-clamp recordings are compiled in Supplementary Table S2 online.

**Figure 7:**
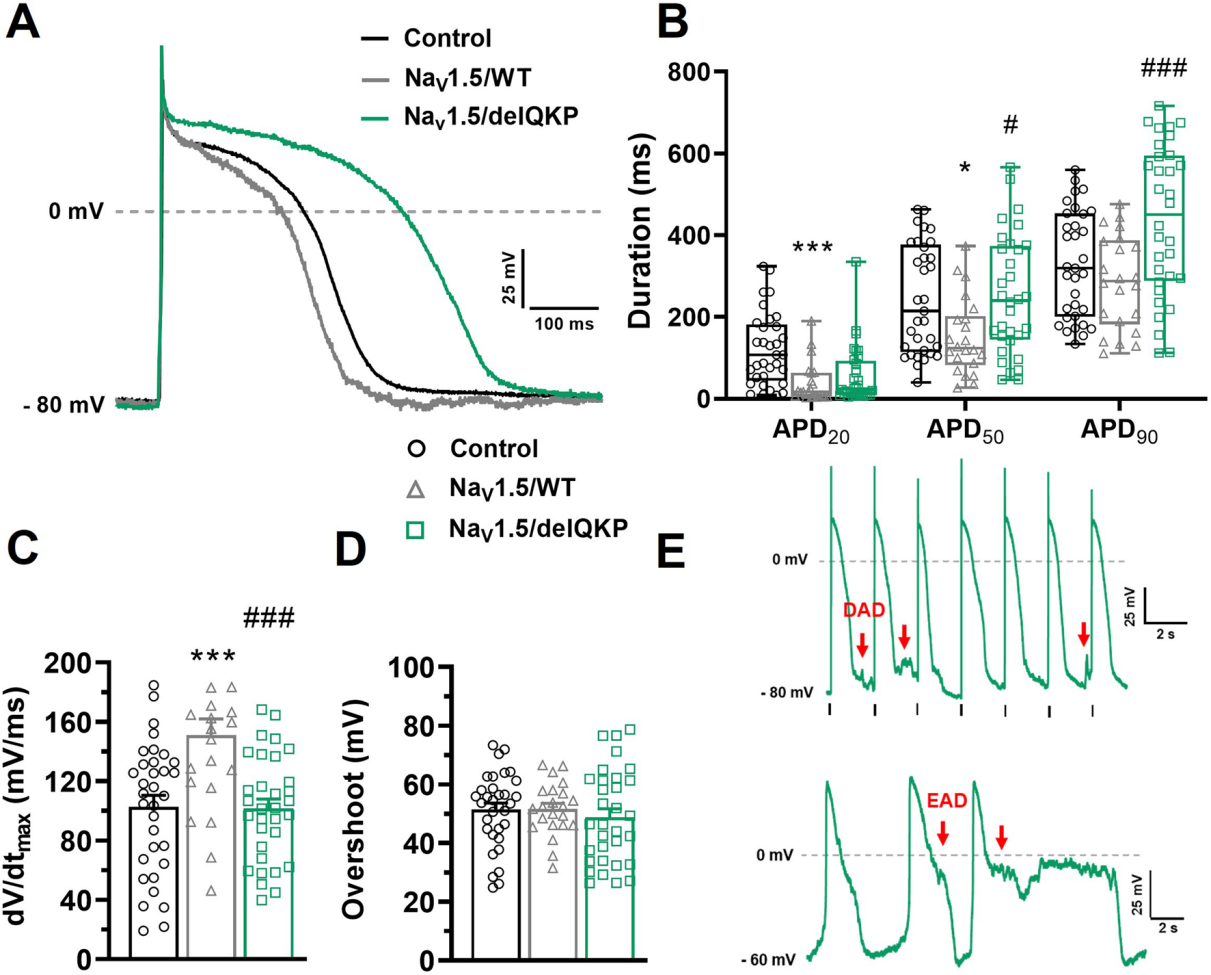
APD prolongation, dV/dt affected and arrhythmogenic events in KO iPSC-CMs expressing Na_V_1.5/delQKP. (**A**) Representative APs traces at a stimulation frequency of 1 Hz recorded in control and KO iPSC-CMs transfected with Na_V_1.5/WT or Na_V_1.5/delQKP. (**B**) Box and whiskers summarizing the median (with quartiles and min to max values) of APD at 20%, 50%, or 90% of repolarization at 1 Hz. *p < 0.05, ***p < 0.001: control versus Na_V_1.5/WT; #*p* < 0.05, ###*p* < 0.001: Na_V_1.5/WT versus Na_V_1.5/delQKP. A nonparametric Kruskal–Wallis test was performed. (**C,D**) Bar graph showing the mean ± SEM of the dV/dt_max_ (**C**) and the overshoot of APs (**D**) recorded at 1 Hz. ***p < 0.001: control versus Na_V_1.5/WT; ###*p* < 0.001: Na_V_1.5/WT versus Na_V_1.5/delQKP. Significance was determined by ANOVA and Dunnett’s post hoc tests. (**E**) Examples of DAD (red arrows) recorded at a stimulation of 0.5 Hz and EAD (red arrows) recorded in spontaneous AP (gap-free mode) in Na_V_1.5/delQKP. Control n = 33, Na_V_1.5/WT n = 22 and Na_V_1.5/delQKP n = 32. This figure was made using MICROSOFT POWERPOINT Version 2106 (build 14131.20278 Office, https://docs.microsoft.com/en-us/) and PRISM 8 Version 8.0.2 (263, https://www.graphpad.com/).

One of the major advantages of our new expression system using Na_V_1.5/KO iPSC-CMs is the ability to study the impact of the mutation on APs directly. The results described above showed that the Na^+^ current was restored but was affected when Na_V_1.5/delQKP was transfected. We measured the impact of these biophysical effects on APs in current-clamp experiments. The first noticeable effect seen on APs after Na_V_1.5/delQKP transfection was the increase in APD compared to those recorded in cells transfected with the WT channel. The representative AP traces in Fig. 7A clearly show this increase in duration for Na_V_1.5/delQKP. The APD_50_ and APD_90_, but not APD_20_, significantly increased by 101 and 150 ms, respectively, in Na_V_1.5/delQKP following 1-Hz stimulations (Fig. 7B). Differences were also observed between control iPSC-CMs and Na_V_1.5/WT iPSC-CMs. The APD_20_ and APD_50_ of Na_V_1.5/WT were 81 and 92 ms smaller, respectively, than those of the control. The dV/dt_max_, which measures the kinetics of the rising phase of APs, was also affected and was significantly slower in Na_V_1.5/delQKP (101.7 ± 6.2 mV/ms) than in Na_V_1.5/WT (151.1 ± 10.9 mV/ms) (Fig. 7C). The dV/dt_max_ of Na_V_1.5/WT was significantly faster than that of the control (102.8 ± 7.7 mV/ms) (Fig. 7C). The AP overshoots were not affected, regardless of the iPSC-CMs studied, and maintained values of approximately 50 mV (Fig. 7D). The above results were determined when the iPSC-CMs were stimulated at 1 Hz. However, the current-clamp recordings were also conducted at stimulation frequencies of 0.5, 1.5, and 2 Hz. When stimulated at 1.5 Hz and above, the cells transfected with Na_V_1.5/delQKP were more often unable to adapt to the rhythm of stimulation. In fact, at 1.5 Hz, 40% (8/20 cells) of the Na_V_1.5/delQKP iPSC-CMs were unable to follow the rhythm compared to only 11% (2/18 cells) for Na_V_1.5/WT. At 2 Hz, 90% (18/20 cells) of Na_V_1.5/delQKP cells were unable to adapt to the rhythm of stimulation. Arrhythmic events such EADs (early afterdepolarizations) and DADs (delayed afterdepolarizations) were also observed on a regular basis in Na_V_1.5/delQKP iPSC-CMs. Figure 7E shows examples of DADs recorded at 0.5 Hz in Na_V_1.5/delQKP cells as well as EADs recorded in spontaneous APs.

## Discussion

In this study, we used CRISPR-Cas9 genomic editing tool to successfully produce a novel iPS cell line in which the *SCN5a* gene coding for the cardiac Na^+^ channel Na_V_1.5 was knocked-out. This iPS cell line was used to produce cardiomyocytes (iPSC-CMs) that no longer express Na_V_1.5 channels. We first characterized by electrophysiological recordings and optical mapping analysis the effect of the Na_V_1.5 KO on the AP itself and their propagation in iPSC-CMs monolayer. Secondly, we re-introduced by transient transfection a WT Na_V_1.5 channel or a Na_V_1.5 LQT3 variant, delQKP, as a proof-of-concept to study Na_V_1.5-linked variants in a human cardiomyocyte environment.

Na_V_1.5 KO iPSC-CMs exhibited spontaneous beats with a frequency comparable to the control cell line. Morphologically, we observed that Na_V_1.5 KO iPSC-CMs express MLC2v and troponin T, two essential structural proteins involved in the formation of myosin and actin filaments, respectively. These proteins play an essential role in the organization of sarcomeres, and in the parallel alignment and movement of the filaments^11, 12^. The suppression of Nav1.5 channels obviously did not affect the organized contractile apparatus to modify the contractility capabilities and rhythm of iPSC-CMs.

As expected, electrophysiological recording revealed a marked 97% reduction in Na^+^ currents in the Na_V_1.5 KO iPSC-CMs. A very small inward and fast-gated Na^+^ current remains. By its high sensitivity to TTX, this inward current could probably be carried by Na_V_1.7 channels which have already been identified by our group as slightly expressed in iPSC-CMs^13^. Such a reduction in Na^+^ currents should have a major impact on the APs given their role in the triggering and the rising phase (phase 0) of the APs. Furthermore, the entry of Na^+^ in the cardiomyocytes is normally followed by a cascade of ion channels activation such as K^+^ and Ca^2+^ channels that contribute to the plateau and the repolarisation phase of APs^14^. We can immediately notice that APs were still elicited in Na_V_1.5 KO iPSC-CMs. Their resistance to TTX (1 µmol/L) but their complete inhibition by nifedipine (1 µmol/L) reveal the Ca^2+^ nature of the APs in the KO cells. These Ca^2+^ APs were characterized by a rising phase (phase 0) considerably slow due to the lack of Na^+^ channel, as calculated by the dV/dt_max_ from the electrophysiological recordings. Moreover, a stronger injected current was required to trigger Ca^2+^ APs. Normally, during the phase 0, Na^+^ channels contribute to more depolarization and the opening of Ca^2+^ channels. In our situation where there are few Na^+^ channels, a stronger depolarization is required to open Ca^2+^ channels.

These alterations will certainly impact the propagation of the electrical signal through the myocardium. We take advantage of the properties of iPSC-CMs to form electrical coupled syncytia of monolayers and we have indeed found a drastically slowing of the conduction velocity in the KO. The role of Na_V_1.5 in the cardiac conduction was already known as shown in a previous study with heterozygous (*Scn5a*^+/−^) Na_V_1.5 mice. These mice exhibited a 50% reduction in Na^+^ current and had impaired atrioventricular conduction and delayed intra myocardial conduction^15^. Our optical mapping experiments confirmed the critical role of Na_V_1.5 for controlling the speed of electrical wave propagation trough myocytes. The slowing of conduction velocity could be related to the lack of Na^+^ channels in the intercalated discs, but, probably to connexins dysregulation, where their role must be studied in the context of Na_V_1.5 KO. It is known that connexins interact physically with Na^+^ channels and the lack of these channels in the intercalated discs may impede their role in establishing a normal conduction velocity.

Another major parameter involved was the duration of the action potential. We found contradictory results according to the experiments carried out. In electrophysiological recordings, the duration of the AP was found to be prolonged while this was shortened in optical mapping analysis in the Na_V_1.5 KO iPSC-CMs. In cardiomyocytes, Na_V_1.5 influence the duration of the AP by the non-inactivating channels that re-open and generate a small inward current (persistent Na^+^ current) during the plateau phase. Previous studies have shown that TTX, a Na^+^ channel blocker, shortened the APD in dog and guinea pig ventricle heart muscle but not in atrial^1, 16^. In addition, heterozygous (*Scn5a*^+/−^) Na_V_1.5 mice showed shorter APDs in the right ventricular epicardium but not in the left ventricular^17^. Studies with other Na^+^ blockers such as flecainide, quinidine and lidocaine showed that the APD can also be increased depending on the stimulation frequency, tissue location and species^17–19^. To resume, the effect of reduction or the suppression of Na_V_1.5 expression on APDs is not straightforward but influenced by the tissue and the Na^+^ channel blocker used, among other factors. In this study, the results disparity between patch-clamp and optical mapping experiments could originate from the nature of the sample. In one case, the recordings were done in individual cells whereas in the other case they were performed on monolayers as behave as a functional syncytium. This highlights the complexity of the contributing factors that can influence the duration of the action potential. Further studies are warranted to understand more this difference.

To complete the Na_V_1.5 KO iPSC-CMs characterization, we were interested to Ca^2+^ homeostasis by measuring the Ca^2+^ currents by the Patch-clamp technique and the propagation of Ca^2+^ transients by optical mapping. The absence of Na_V_1.5 does not modify the biophysical properties of L-type Ca^2+^ channels but altered the propagation of Ca^2+^ transients. Ca^2+^ transients represent Ca^2+^ release/reuptake cycles that coordinate contraction and relaxation of cardiomyocytes. Even though Na_V_1.5 KO iPSC-CMs beat spontaneously and at rhythm similar to control, Ca^2+^ propagation velocity has significantly decreased. This result is perfectly correlated with the reduction in conduction velocity and could be explained by alteration of the excitation–contraction coupling in the absence of Na_V_1.5 in cardiomyocytes. The intracellular Ca^2+^ release is triggered by the AP, which their conduction is delayed in the KO, which therefore also causes the observed delay in the wave of Ca^2+^ release. Optical recordings on Na_V_1.5 KO iPSC-CMs also showed a decrease in amplitude of Ca^2+^ transients signal, probably indicating a reduction of Ca^2+^ availability at each cycle. This could be explained by other Ca^2+^ handling players such as Ryanodine channel or SERCA pump.

In the second part of this study, we have re-introduced by DNA transfection the Na_V_1.5 WT channel or the Na_V_1.5 delQKP LQT3 variant in the Na_V_1.5 KO iPSC-CMs and compared their biophysical effects. The transfection of the Na_V_1.5/WT channel in KO iPSC-CMs successfully restored Na^+^ channel expression and the fast upstroke velocity of APs. In fact, the upstroke velocity was even faster in Na_V_1.5/WT than in control. We hypothesize that this difference could be explain be the higher level of Nav1.5 channels due to its overexpression produced by the plasmid transfection.

*SCN5a* mutations leading to LQT3 are generally associated with a gain of channel function, characterized by an increase in the persistent Na^+^ current leading to delayed repolarization and sustained APD. DelQKP is the deletion of three amino acids (QKP) at positions 1507–1509 on Na_V_1.5^20^. This mutation is located in the DIII–DIV linker region of Na_V_1.5, which is essential in the inactivation process of the channel^21^. This variant has already been characterized by using non-cardiac cells and heterozygote knock-in delQKP *Scn5a*^Δ/+^ mice^20, 22^. In the present study, our approach was to use iPSC-CMs in order to provide a human and a native cardiac cell environment to evaluate and validate the effects of the Na_V_1.5 variant. Our results in iPSC-CM confirmed that the QKP deletion cause a destabilization of the inactivated state of the channel, that is: a hyperpolarized shift of the inactivation, a faster inactivation decay time, a faster reactivation and finally an increased persistent Na^+^ current. We obtained similar biophysical alterations of the channel that those reported previously in non-cardiac cells (tsa201 cells) and in cardiomyocytes from delQKP *Scn5a*^Δ/+^ mice^20, 22^.

The strength of our model lies in the possibility of recording cardiac APs and measuring the effect of Na_V_1.5 variants. We show for the first time that iPSC-CMs transfected with Na_V_1.5/delQKP exhibit the essential LQT3 features. Their APs presented a delayed and a prolonged repolarization phase, leading to arrhythmic events such as EADs and DADs. The prolongation of the AP is most likely caused by the increase in the persistent Na^+^ current seen in voltage-clamp recordings The continuous entry of Na^+^ into the cell keeps the Ca^2+^ channels activated and increases the release of Ca^2+^ from the sarcoplasmic reticulum^23^. As consequence, the duration of the phase 2 of the AP increases. Moreover, the increase in APD may be caused also by the increased rate of Na_V_1.5 recovery after inactivation as demonstrated for the I1768V variant^24^. The arrhythmic events caused by the lengthening of the AP were previously reported in the delQKP *Scn5a*^Δ/+^ mouse model. Therefore, our new-approach using Na_V_1.5 KO iPSC-CMs is capable of reproducing what seen in mouse model but in cardiomyocytes-like of human origins.

An increase in the duration of APs at the cellular level results in a prolongation of the QT interval on the electrocardiogram of patients with LQT syndrome. Moreover, patients are at increased risk for the development of polymorphic ventricular tachycardia, specifically torsade de pointes (TdP)^25^. Thanks to our model, we were able to show that the delQKP variant induces a prolongation of APD at the origin of LQT, and more interesting, causes EADs, responsible for TdP^26^.

Interestingly, during electrophysiological recordings, we observed differences between the control and Na_V_1.5 KO iPSC-CMs transfected with Na_V_1.5/WT channel. Indeed, the Na^+^ current exhibited a hyperpolarized threshold of activation, faster kinetic of inactivation and slower recovery from inactivation compared to control cells (iPSC-CMs not knocked-out for Na_V_1.5). iPSC-CM transfected with Na_V_1.5/WT also generated APs with a faster depolarization phase and a shortened repolarization phase (APD_20_ and APD_50_ only). As mentioned earlier, these differences may be caused by channel overexpression on iPSC-CMs. But interestingly, it could also be explained by the differences between the adult and the fetal isoform of Na_V_1.5^27^. Although the efficiency of iPSC differentiation to cardiomyocytes has increased considerably with novel differentiation protocols, the level of iPSC-CM maturity remains an issue. This low level of maturity is characterized by the expression of numerous fetal isoforms to the detriment of adult isoforms, including those of Na_V_1.5^10, 28^. Thereby, when we compared the transfected Na_V_1.5/WT with the control iPSC-CMs, in fact we mostly compared the effects between the adult and the fetal isoforms. However, when we compared the transfected Na_V_1.5/WT with the transfected Na_V_1.5/delQKP, both channels were in the adult isoform background. This is an additional advantage of the new Na_V_1.5 KO iPSC-CM, i.e., the ability to study Na_V_1.5 variants in the desired background, either adult or fetal isoforms.

## Conclusion

In conclusion, thanks to our Na_V_1.5 KO iPSC model, we were able first to characterize the impact of the KO of Na_V_1.5 on APs as well as on the propagation of voltage and Ca^2+^ waves on iPSC-CMs. Secondly, as a proof-of-concept, we were able to directly study the impacts on the APs of a known mutation linked to LQT3. The workflow of our novel approach is straightforward. The iPSC-CM from the Na_V_1.5 KO cell line can be frozen and thawed on demand to study any Na_V_1.5 variants by a simple transfection, as easily than with an expression system such as HEK293 cells with however several advantages as described above. Na_V_1.5 variants and drug effects can now be straightforwardly and easily studied in a cardiac cellular environment. We hope that in the future, our new tool will help in the diagnosis and treatment of patients suffering of cardiovascular diseases.

## Supplementary Information

Supplementary Video 1.

Supplementary Video 2.

Supplementary Video 3.

Supplementary Video 4.

Supplementary Information 1.

## Abbreviations

AP: Action potential
APD: Action potential duration
CRISPR/Cas9: Clustered regularly interspaced short palindromic repeats/associated protein 9
CHO: Chinese hamster ovary
CICR: Calcium-induced calcium release
cTnT: Cardiac troponin T
DAD: Delayed afterdepolarization
DelQKP: Deletion of three amino acids QKP (glutamine, lysine, proline)
dV/dt_max_: Upstroke velocity
EAD: Early postdepolarization
HEK: Human embryonic kidney
iPSC-CM: Cardiomyocytes derived from induced pluripotent stem cells
KO: Knock-out
LQT3: Long QT syndrome type 3
Mlc2v: Ventricular myosin light chain 2
OM: Optical mapping
SCN5a: Sodium voltage-gated channel alpha subunit 5
TdP: Torsade de pointes
TTX: Tetrodotoxin
VGSC: Voltage-gated sodium channel
WT: Wild type
