# Supplementary material for: NaV1.5 knockout in iPSCs: a novel approach to study NaV1.5 variants in a human cardiomyocyte environment
Source: Sci Rep. 2021 Aug 25;11:17168. doi: 10.1038/s41598-021-96474-6 (PMC8387439; doi:10.1038/s41598-021-96474-6)
Supplement: Supplementary file 5 — Supplementary Information 1. [file 41598_2021_96474_MOESM5_ESM.docx]

**Na_V_1.5 knockout in iPSCs: a novel approach to study Na_V_1.5 variants in a human cardiomyocyte environment**

Marion Pierre^1^, Mohammed Djemai^1^, Hugo Poulin^1^, and Mohamed Chahine^1,2^

^1^CERVO Brain Research Center, Quebec City, QC, Canada

^2^Department of Medicine, Faculty of Medicine, Université Laval, Quebec City, QC, Canada

**Supplementary Information**

Supplementary Figure S1

Supplementary Figure S2

Supplementary Figure S3

Supplementary Figure S4

Supplementary Figure S5

Supplementary Figure S6

Supplementary Figure S7

Supplementary Table S1

Supplementary Table S2

Legends for video files

Supplementary Videos S1-S4

**
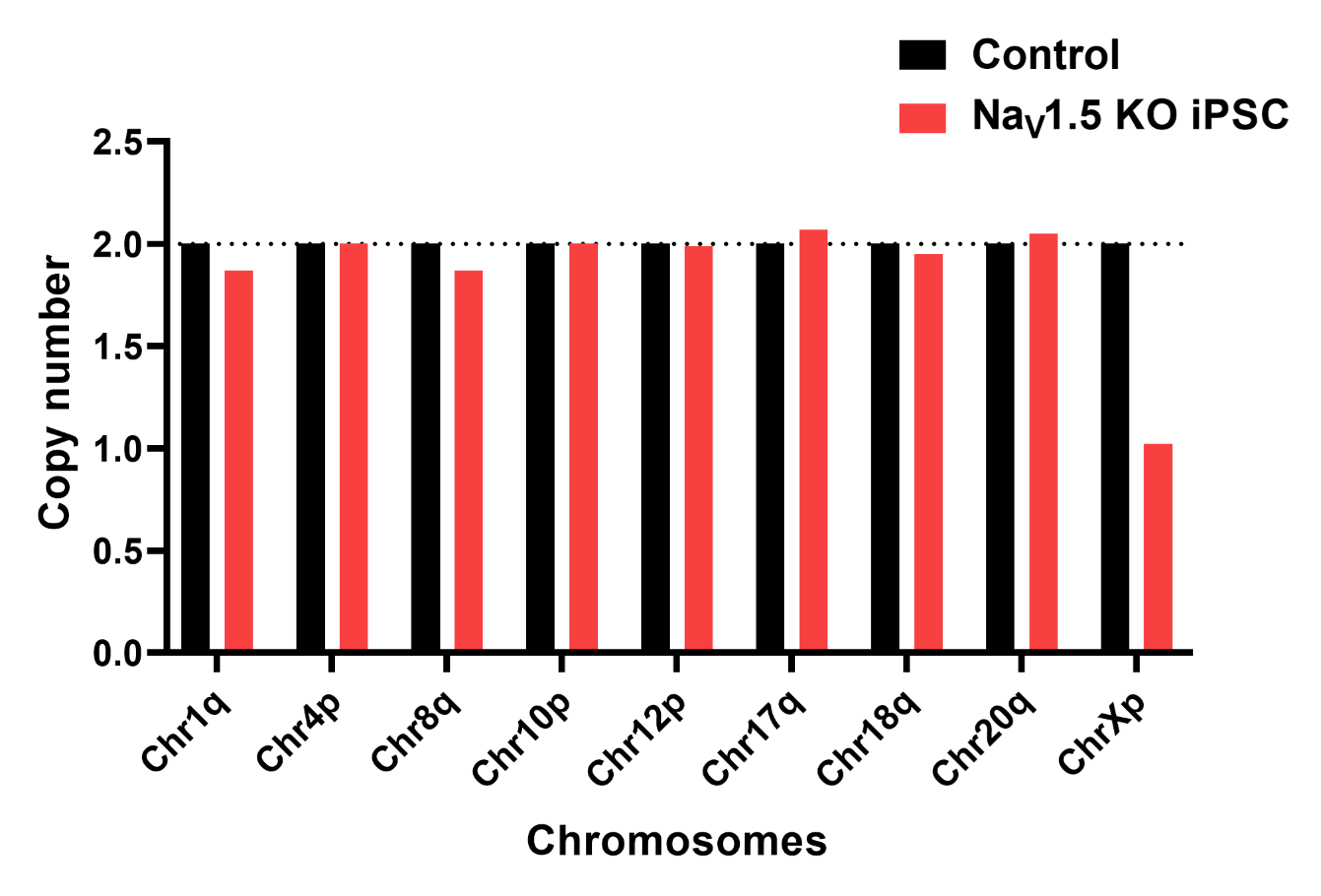
Supplementary Figure S1. Chromosome’s status of Na_V_1.5 KO iPSCs.** The histogram represents the copy number of 8 regions. The genetic screening was performed by quantitative PCR (qPCR) using the hPSC Genetic Analysis Kit (Cat #07550, StemCell Technologies, BC, Canada). The kit includes a genomic DNA control with normal copy number over the 8 regions. This figure was made using GraphPad PRISM 8 Version 8.0.2 (263, https://www.graphpad.com/).


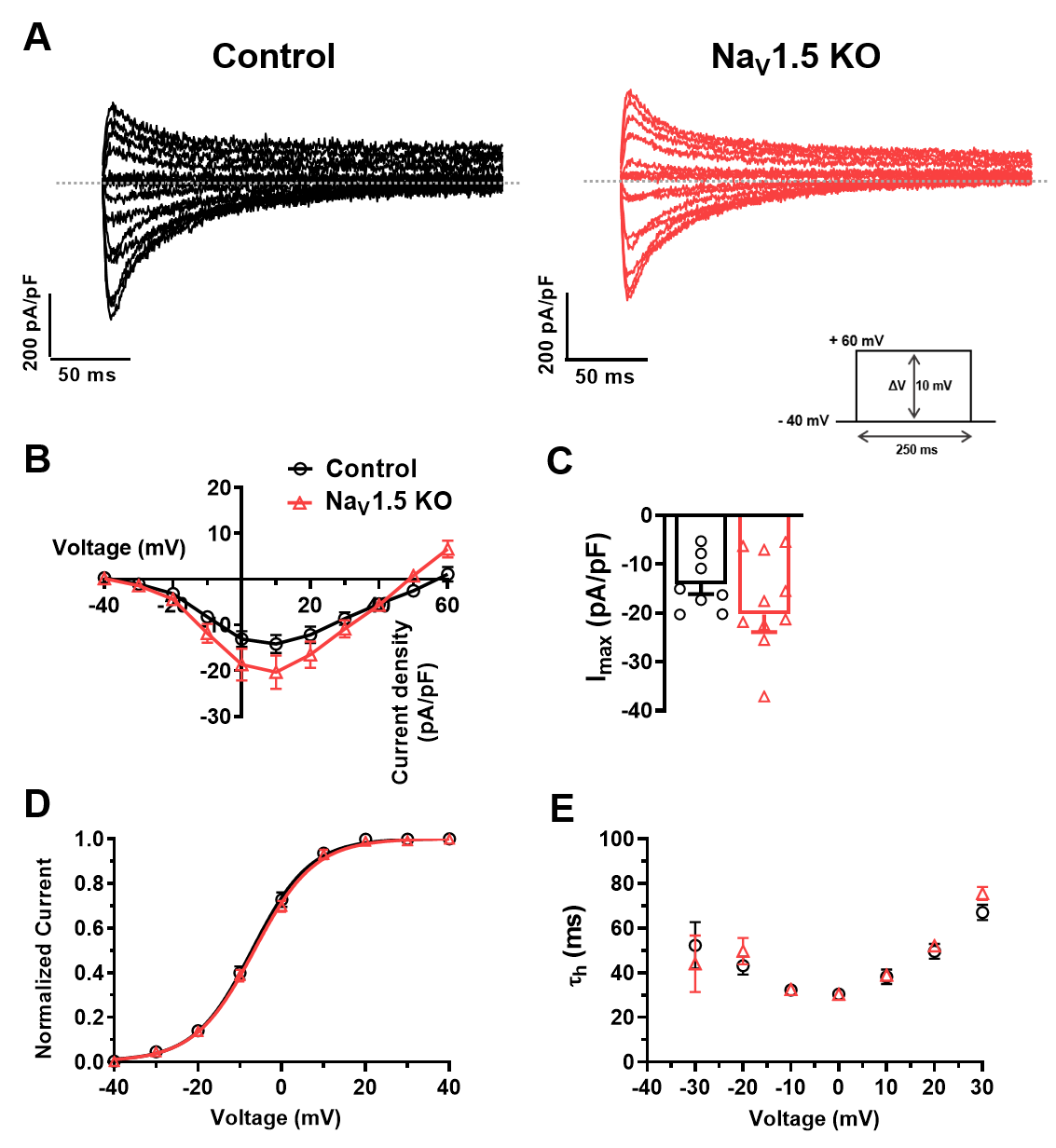
**Supplementary Figure S2. Biophysical properties of Ca^2+^ channel in Na_V_1.5 KO iPSC-CMs.** **(A)** Representative Ca^2+^ currents recorded in control and Na_V_1.5 KO iPSC-CM. The dashed line represents zero current. The internal solution was composed of (mM): 25 NaCl, 105 CsCl, 1 MgCl_2_, 10 EGTA, and 10 HEPES. The pH was adjusted to 7.2 with 1N CsOH. The external solution used was composed of (mM): 100 NaCl, 5 CsCl, 5 CaCl_2_, 40 NMDG, 1 MgCl_2_, 10 D-glucose, 10 HEPES. and 15 TEA-Cl. The pH adjusted to 7.4 with 1N methanesulfonic acid (MSA). **(B)** L-type Ca^2+^ channel current-voltage relationships recorded in control (n = 8) and Na_V_1.5 KO iPSC-CMs (n = 11). The current was normalized to the capacitance (pF) of the cells. **(C)** Dot plot showing the L-type Ca^2+^ channel current densities recorded at 10 mV. (**D**) Voltage-dependence of steady-state activation inactivation of L-type channel Ca^2+^ currents. (**E**) The time constants of fast inactivation decay were plotted as a function of voltage for the control and Na_V_1.5 KO iPSC-CMs. The time constants were obtained using a single exponential function: (A(exp(−t/τ) + C). Bars indicate SEM. This figure was made using MICROSOFT POWERPOINT Version 2106 (build 14131.20278 Office, https://docs.microsoft.com/en-us/) and PRISM 8 Version 8.0.2 (263, https://www.graphpad.com/).


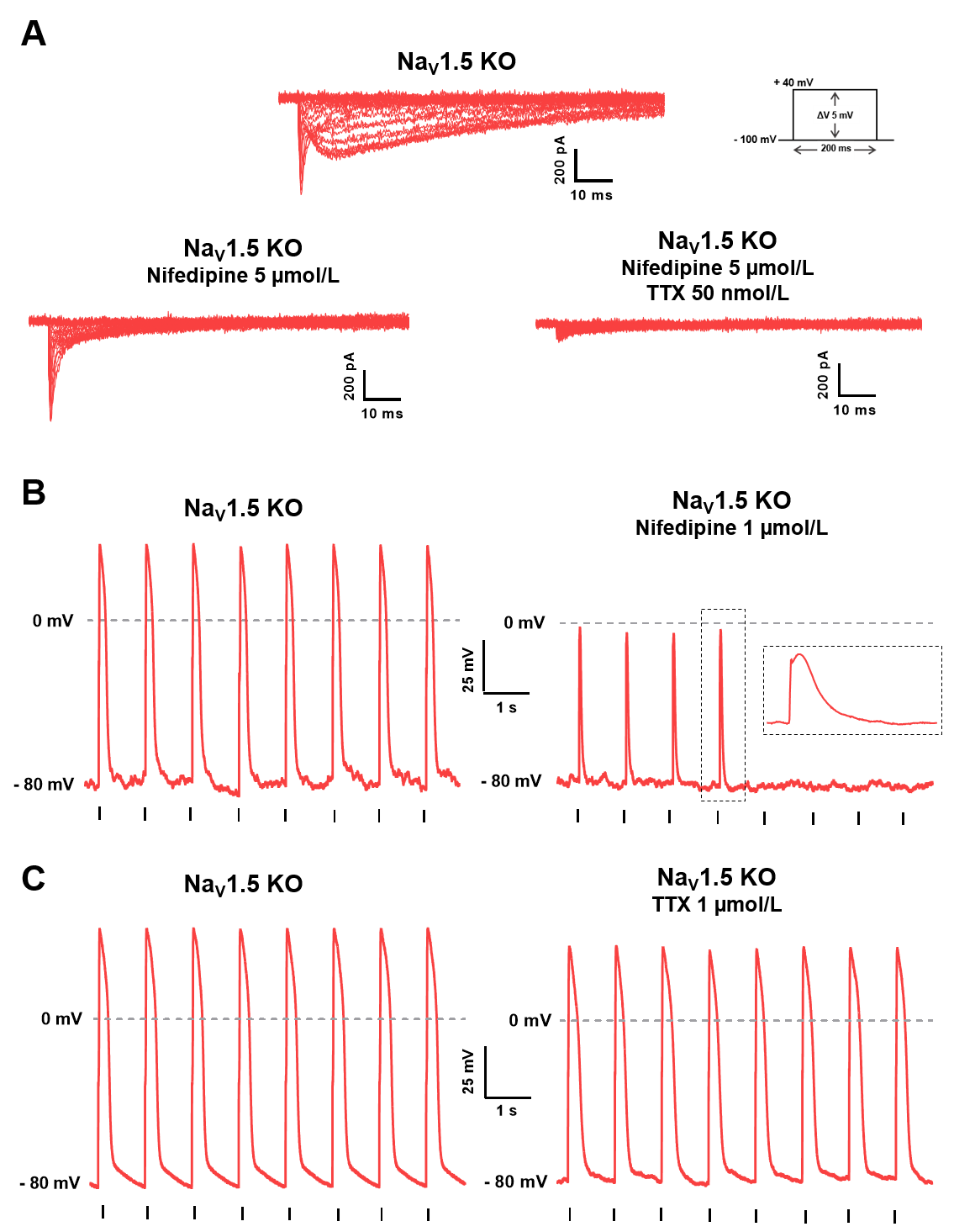
**Supplementary Figure S3. Pharmacology of Na_V_1.5 KO iPSC-CMs.** **(A)** Representative Na^+^ and Ca^2+^ currents recorded in Na_V_1.5 KO iPSC-CM (upper panel), following a 5µmol/L nifedipine treatment (bottom left panel) and after the addition of 50 nmol/L TTX (bottom right panel). The three recordings come from the same cell. **(B)** APs traces recorded in Na_V_1.5 KO iPSC-CMs at a stimulation frequency of 1 Hz before (left panel) and after (right panel) 1µmol/L nifedipine treatment. The dotted square is a zoom-in on one AP. **(C)** APs traces recording in Na_V_1.5 KO iPSC-CMs at a stimulation frequency of 1 Hz before (left panel) and following 1 µmol/L TTX treatment (right panel). This figure was made using MICROSOFT POWERPOINT Version 2106 (build 14131.20278 Office, https://docs.microsoft.com/en-us/) and PRISM 8 Version 8.0.2 (263, https://www.graphpad.com/).

**
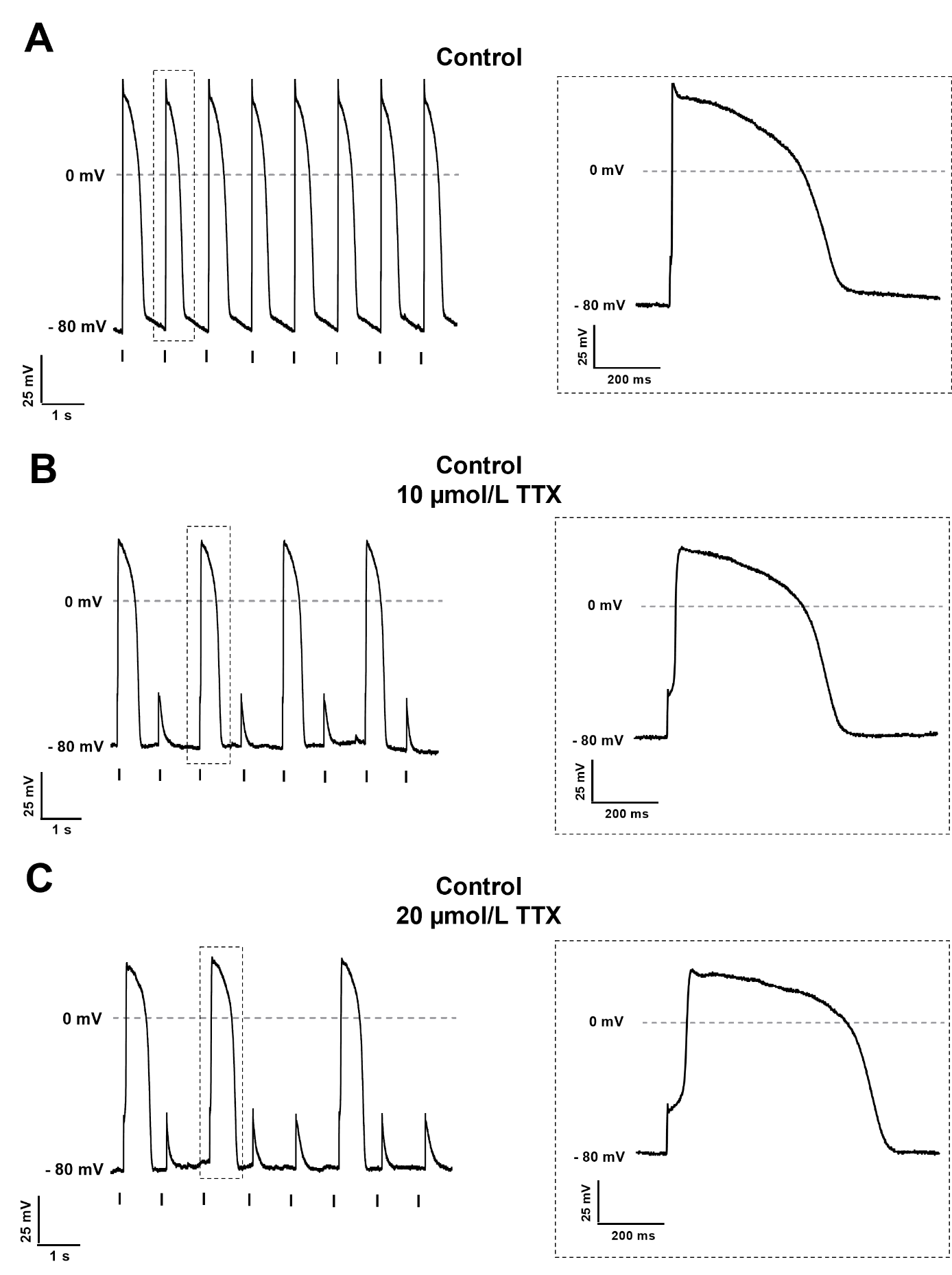
Supplementary Figure S4. Effect of Na^+^ channel inhibitor on APs from control iPSC-CMs.** **(A)** APs traces recorded in control iPSC-CMs at a stimulation frequency of 1 Hz. The dotted square is a zoom-in on one AP. **(B)** and **(C)** and represent APs traces recorded in control iPSC-CMs after 10 µmol/L and 20 µmoL/L TTX concentrations, respectively. The dotted square is a zoom-in on one AP. The four recordings come from the same cell. This figure was made using MICROSOFT POWERPOINT Version 2106 (build 14131.20278 Office, https://docs.microsoft.com/en-us/) and PRISM 8 Version 8.0.2 (263, https://www.graphpad.com/).


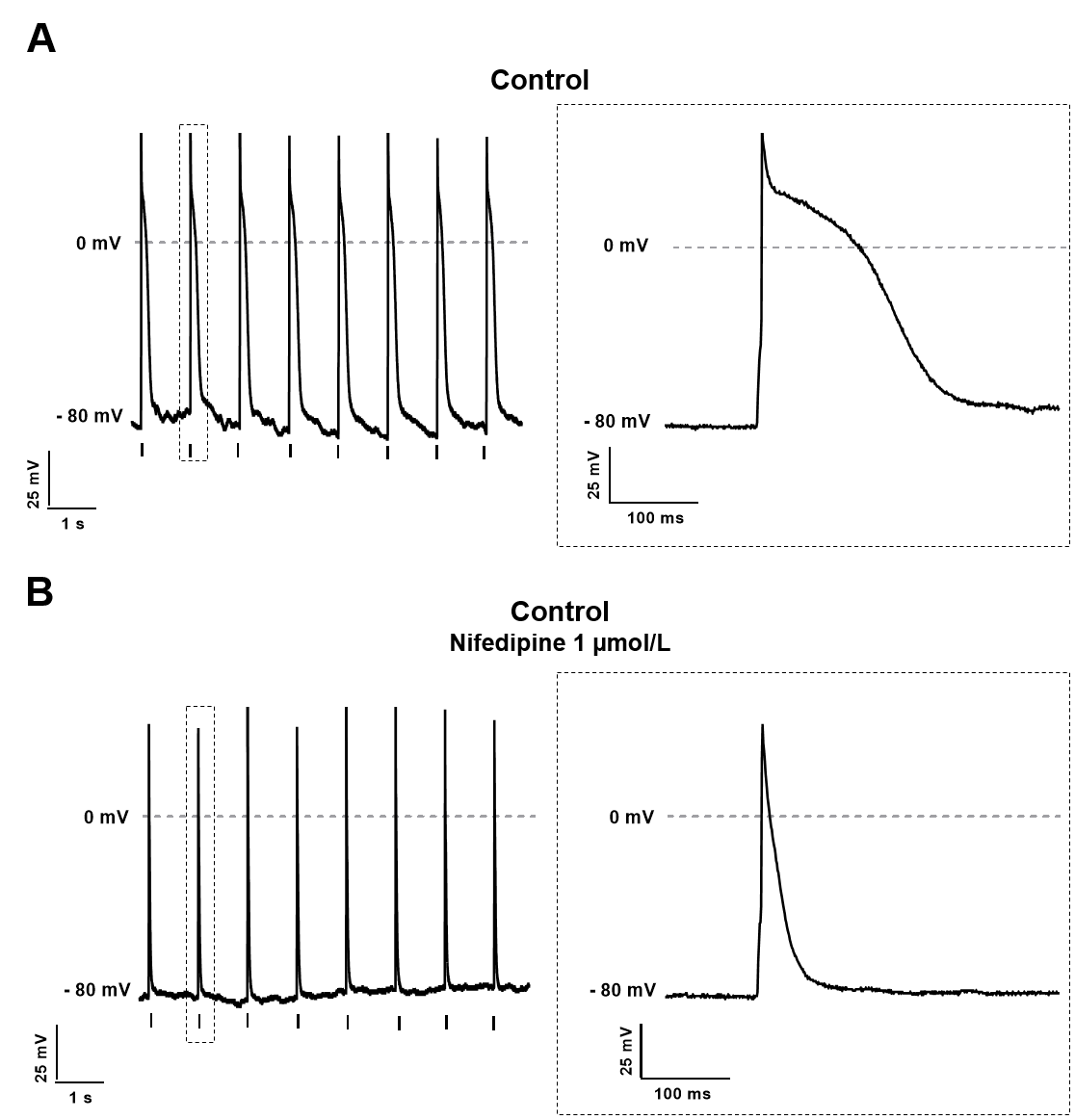
**Supplementary Figure S5. Effect of Ca^2+^ channel inhibitor on APs from control iPSC-CMs.** **(A)** APs traces recorded in control iPSC-CMs at a stimulation frequency of 1 Hz. **(B)** APs traces recorded in control iPSC-CMs after 1µmol/L nifedipine treatment. The dotted square is a zoom-in on one AP. The two recordings come from the same cell. This figure was made using MICROSOFT POWERPOINT Version 2106 (build 14131.20278 Office, https://docs.microsoft.com/en-us/) and PRISM 8 Version 8.0.2 (263, https://www.graphpad.com/).


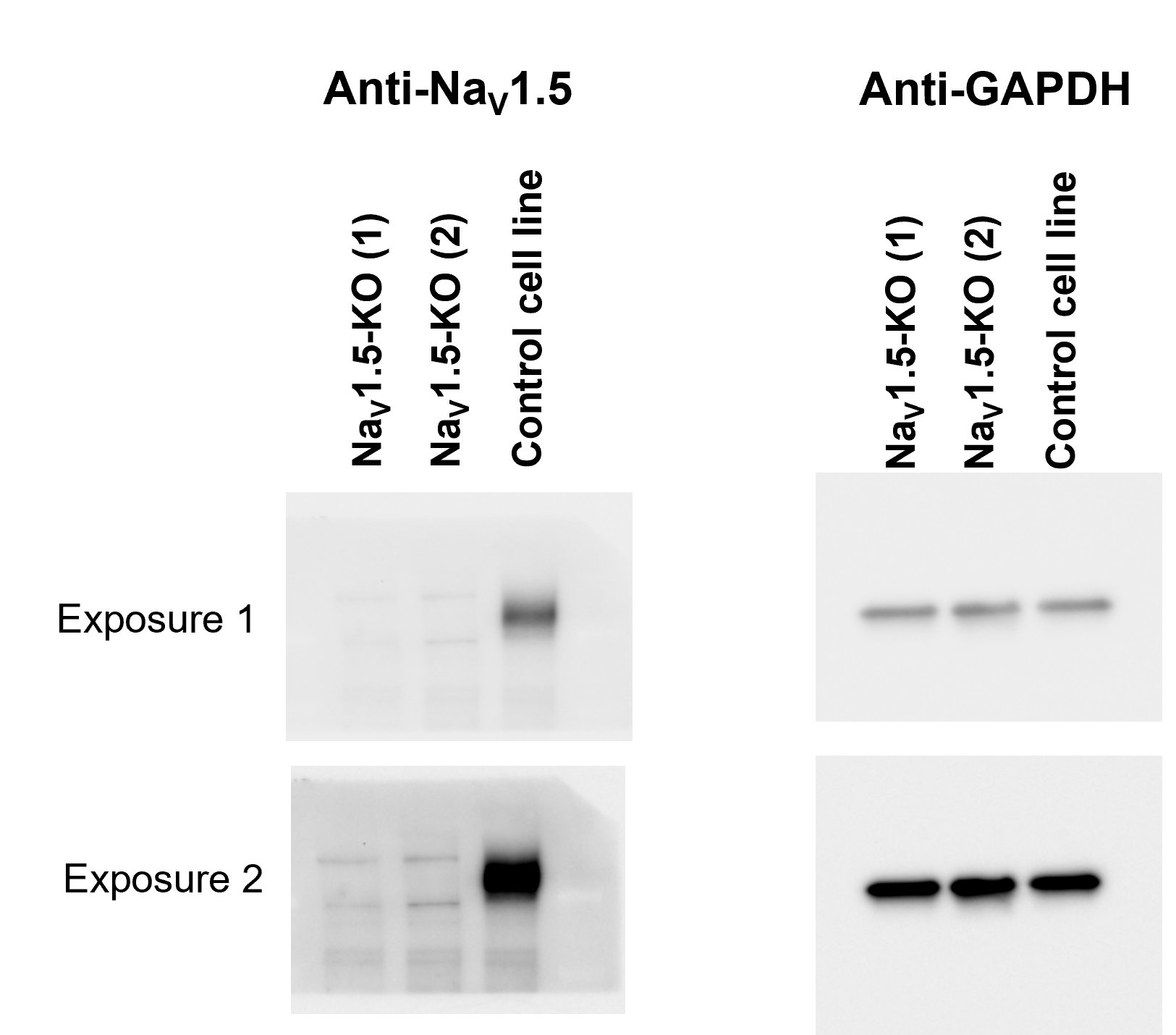
**Supplementary Figure S6. Unedited blots for anti-Na_V_1.5 and anti-GAPDH antibodies.** The Western blots were performed on protein extracts from two independent Na_V_1.5 KO differentiations compared to one control cell line. These two blots come from same gel. The membranes were cut in two parts. One part was blot with the anti-Na_V_1.5. The other part was blot with anti-GAPDH antibody, used as a loading control. The protein was revealed using the stain-free technology from BioRad. This figure was made using MICROSOFT POWERPOINT Version 2106 (build 14131.20278 Office, https://docs.microsoft.com/en-us/) and IMAGEJ 1.52i (Java 1.8.0_66, http://imagej.nih.gov/ij).


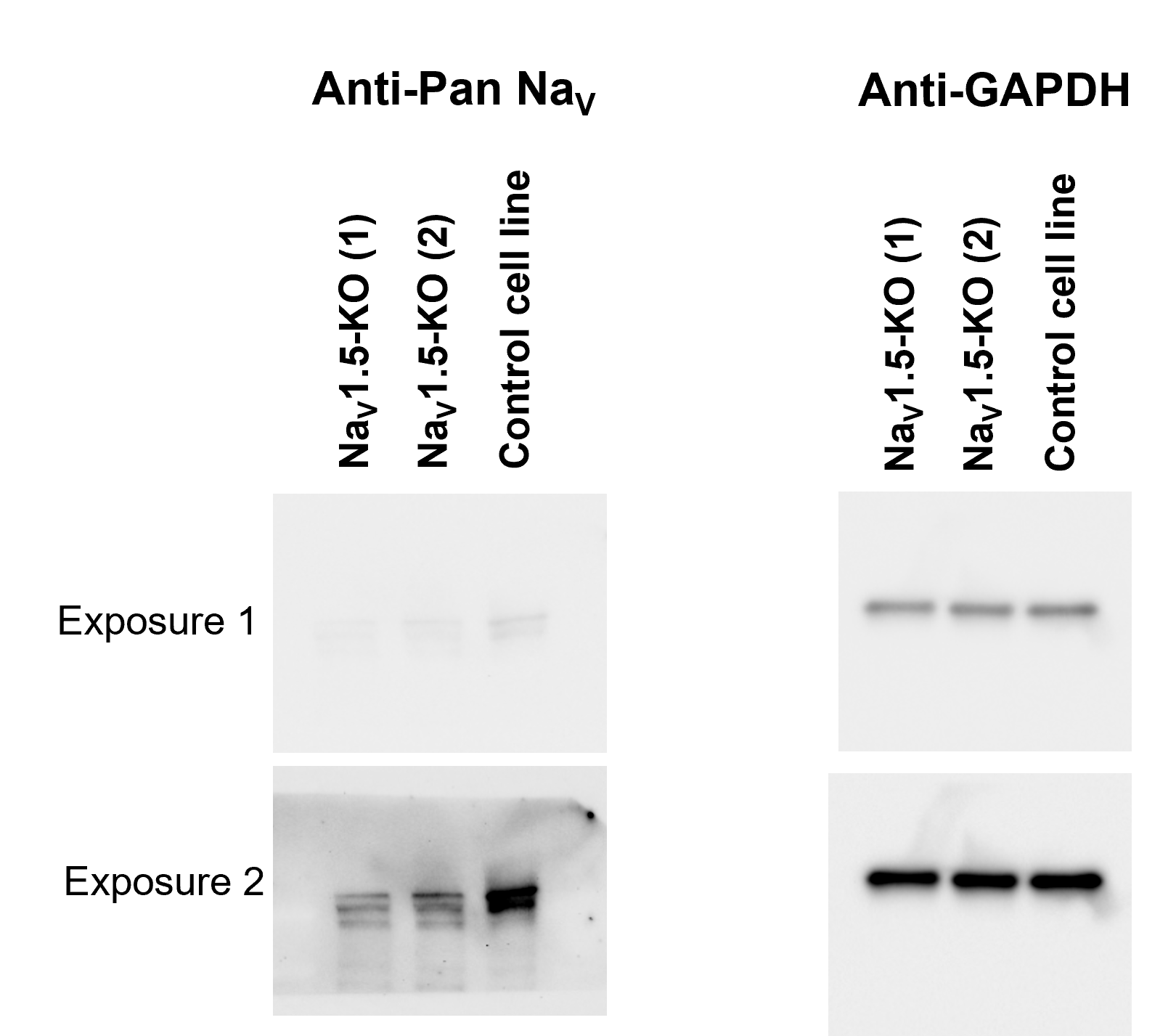
**Supplementary Figure S7. Unedited blots for anti-Pan Na_V_ and anti-GAPDH antibodies.** The Western blots were performed on protein extracts from two independent Na_V_1.5 KO differentiations compared to one control cell line. These two blots come from same gel. The membranes were cut in two parts. One part was blot with the anti-Pan Na_V_. The other part was blot with anti-GAPDH antibody, used as a loading control. The protein was revealed using the stain-free technology from BioRad. This figure was made using MICROSOFT POWERPOINT Version 2106 (build 14131.20278 Office, https://docs.microsoft.com/en-us/) and IMAGEJ 1.52i (Java 1.8.0_66, http://imagej.nih.gov/ij).

**Supplementary Table S1.** **Off-target sites of sgRNAs using for CRISPR editing.**

A lower score indicates higher potential editing. #MM indicates the number of mismatches in the gRNA design compared to the off-target site. PAM: protospacer adjacent motif; sgRNA: small guide ribonucleic acid; *SCN5A*: Sodium Voltage-Gated Channel Alpha Subunit 5; *PPHLN1*: periphilin-1; *HOXD13*: Homeobox D13; *LRP1B*: low-density lipoprotein receptor-related protein 1B; *RBM11*: RNA Binding Motif Protein 11; *PARN*: Poly(A)-Specific Ribonuclease; *RAP2B*: Ras-related protein Rap-2b; *ZCCHC18*: Zinc finger CCHC-type containing 18; *TTN*: titin. Chr: chromosome.

**Supplementary Table S2. Parameters of Na_V_1.5 biophysical properties.**

V_1/2_, midpoint for activation or inactivation (mV); k, slope factor for activation or inactivation; τ, time constant (ms); n, number of cells recorded. Values are means ± SEM, *P<0.05, **P<0.01: control *vs* Na_V_1.5/WT. #P<0.05, ##P<0.01: Na_V_1.5/WT *vs* Na_V_1.5/delQKP. Significance was determined by one-way ANOVA with Dunnet’s post hoc test.

**Legends for the Video files**

**Supplementary Video S1: iPSC-CMs in monolayer from control.**Video of iPSC-CMs in monolayer of the control taken in phase contrast. The field of view is 1.42 mm x 1.06 mm. The video is display at normal speed.

**Supplementary Video S2: iPSC-CMs in monolayer from Na_V_1.5 KO.**Video of iPSC-CMs in monolayer of the Na_V_1.5 KO taken in phase contrast. The field of view is 1.42 mm x 1.06 mm. The video is display at normal speed.

**Supplementary Video S3**: **Optical action potential propagation in Control and Na_V_1.5 KO iPSC-CMs monolayers.** Slower conduction velocity (CV) of depolarization wave in Na_V_1.5 KO iPSC-CMs monolayer (left) compared to Control (right) when paced at 1 Hz. Dynamic optical mapping movies are displayed at 60 frames/second, with color bar indicating the normalized fluorescence intensities (ΔF/F_0_).

**Supplementary Video S4**: **Calcium wave spreading** **within Control and Na_V_1.5 KO iPSC-CMs monolayers.** Significantly slower Ca^2+^ transient propagation velocity (CaPV) Na_V_1.5 KO iPSC-CMs monolayer (left) in comparison with Control (right) when paced at 1 Hz. Dynamic optical mapping movies are displayed at 60 frames/second, with color bar indicating the normalized fluorescence intensities (ΔF/F_0_).
